# Songbird species that display more-complex vocal learning are better problem-solvers and have larger brains

**DOI:** 10.1126/science.adh3428

**Published:** 2023-09-14

**Authors:** Jean-Nicolas Audet, Mélanie Couture, Erich D. Jarvis

**Affiliations:** 1 The Rockefeller University Field Research Center, Millbrook, NY, USA.; 2 Laboratory of Neurogenetics of Language, The Rockefeller University, New York, NY, USA.; 3 The Vertebrate Genome Laboratory, The Rockefeller University, New York, NY, USA.; 4 Howard Hughes Medical Institute, The Rockefeller University, New York, NY, USA.

## Abstract

Complex vocal learning, a critical component of human spoken language, has been assumed to be associated with more-advanced cognitive abilities. Tests of this hypothesis between individuals within a species have been inconclusive and have not been done across species. In this work, we measured an array of cognitive skills—namely, problem-solving, associative and reversal learning, and self-control—across 214 individuals of 23 bird species, including 19 wild-caught songbird species, two domesticated songbird species, and two wild-caught vocal nonlearning species. We found that the greater the vocal learning abilities of a species, the better their problem-solving skills and the relatively larger their brains. These conclusions held when controlling for noncognitive variables and phylogeny. Our results support a hypothesis of shared genetic and cognitive mechanisms between vocal learning, problem-solving, and bigger brains in songbirds.

Spoken language and problem-solving are often considered to be components of intelligence in humans. An essential and specialized component of spoken language is vocal production learning, or the ability to imitate sounds (1, 2). Advanced vocal learning has been found in only a handful of taxa, including five mammalian (humans, elephants, cetaceans, pinnipeds, and bats) and three avian (songbirds, parrots, and hummingbirds) clades (1). Interestingly, the vocal learning taxa that display the most-complex vocal learning behavior overlap those long thought to exhibit more-intelligent cognitive capacities [e.g., humans, cetaceans, elephants, corvid songbirds, and parrots (3, 4)], although this has not been quantitatively tested across species.

The few studies that have tested for relationships between vocal learning complexity and cognitive abilities have all been within species (5). Some studies found positive relationships (6, 7), whereas others did not (8–10), and some found negative relationships (7, 11), which led Searcy and Nowicki (5) to conclude that there is not good evidence of such relationships. The challenge for these within-species studies is that it is difficult to tease out whether individual differences are due to genetic factors or cultural or life-experience factors, such as food deprivation (12) or stress (13).

Although vocal learning is often thought of as a dichotomous trait, considerable variation in phenotypes has been documented within lineages, with songbirds being the most speciose and best-characterized clade (1, 14–16). Features that are believed to reflect more-complex vocal learning in songbirds include (i) large vocal repertoires, (ii) lifelong open-ended vocal learning ability, and (iii) mimicry of other species’ vocalizations (16–18). It is unknown whether these vocal learning variations are linked with other cognitive phenotypes.

Cognition has been measured in the laboratory using a variety of behavioral tasks. Of these, problem-solving is assumed to be one of the most complex, requiring animals to figure out the best action to overcomea challenge. Problem-solving may be relevant for allowing animals to cope with ecological disturbances (19). Other cognitive assays that are often conceptually linked with intelligence include associative learning, reversal learning, and self-control tasks (20).

In this work, we quantitatively tested whether there is a linkage between vocal learning complexity and other cognitive traits. We tested 214 individuals from 23 species consisting of 21 wild-caught and two domesticated avian species; 21 species were vocal learning songbirds, and two were vocal nonlearning species (Fig. 1A). We generated a database of vocal learning characteristics of these species from an extensive literature of 80 publications (table S1), assessed species’ performance on a battery of behavioral tasks (fig. S1 and supplementary methods), and found across-species relationships between vocal learning complexity, problem-solving, and relative brain size.

## Vocalization repertoire size is associated with open-endedness and mimicry

Wild birds were caught at the Rockefeller University Field Research Center in New York, USA, over a 3-year period, and domesticated species were bred at the same location (zebra finches) or purchased from a local breeder (canaries). Birds were opportunistically captured in mist nets, and species were chosen if their local abundance allowed for a sufficient sample size (*n* ≥ 12) of males (the vocal learning sex for most species); in addition, for eight species, we kept and tested one or two animals each to see whether they would follow a trend. All birds were habituated individually in cages for 3 days, where they could hear but not see other individuals.

For all 23 species, literature exists on their vocal behavior (table S1). We used these data to establish a consistently defined profile for each species in a database that included six vocal learning characteristics: (i) presence of vocal learning, (ii) open-ended versus closed-ended vocal learning, (iii) capacity for vocal mimicry of other species, (iv) song repertoire size per individual, (v) call repertoire size per species, and (vi) total repertoire size of both songs and calls (supplementary methods and table S1).

**Fig. 1.**
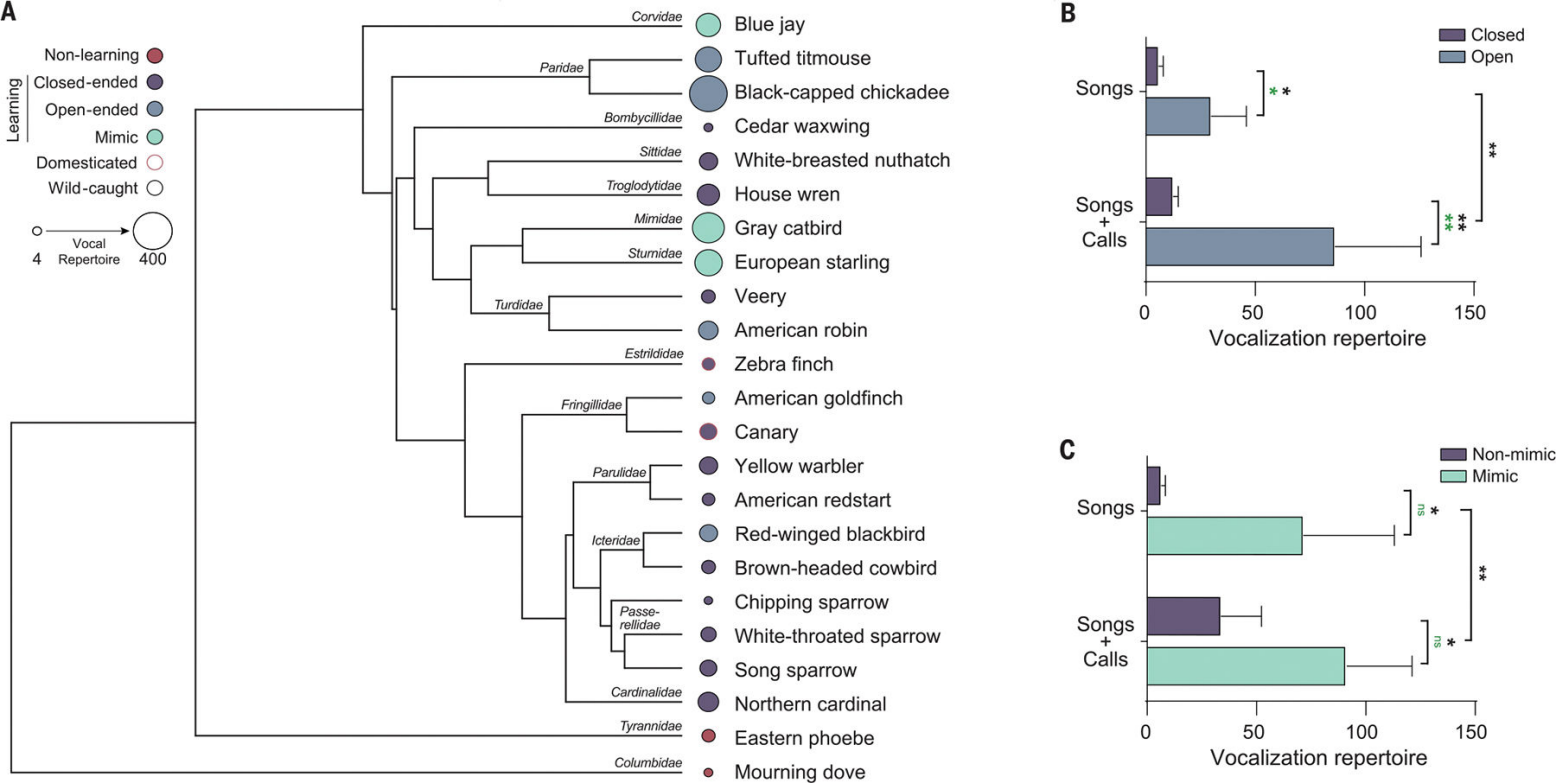
The 23 study species of birds and their vocal learning characteristics. (**A**) Phylogenetic tree of the species that were studied based on (42). Inside circle colors indicate the type of vocal learning, perimeter circle colors indicate domesticated versus wild species, and circle size is proportional to vocalization repertoire size. (**B**) Comparisons of song and call repertoire sizes in open- versus closed-ended vocal learning songbirds (*n* = 21 species). (C) Comparison of mimic and nonmimic species. Black * and ** indicate significant *P*_ANOVA_ at <0.05 and <0.01, respectively. Green * and ** indicate significant *P*_AOV.PHYLO_ (ANOVA accounting for phylogenetic relationships). ns, not significant. Full statistical values are provided in table S2. Bars are means ± SEM.

We found that, consistent with Robinson *et al*. (18), open-ended vocal learning songbirds had significantly larger song repertoires than closed-ended vocal learners (Fig. 1B and table S2A). Using the complete vocalization repertoires (song and calls) yielded an even greater difference between open- and closed-ended vocal learning species (Fig. 1B and table S2A). Similarly, songbird species that are capable of vocal mimicry had larger repertoires than nonmimics (Fig. 1C and table S2A). All of these differences increased in significance when including the two vocal nonlearners (the mourning dove and suboscine eastern phoebe; table S2B), which were at the lower end of the distribution for song and call repertoire sizes (table S1). Nearly all differences were still significant when accounting for phylogenetic relationships in the analysis of variance (ANOVA) model, except for repertoire size between mimic and nonmimic songbirds, which still approached significance [phylogenetic ANOVA *P* value (*P*_AOV.PHYLO_) ~ 0.07; table S2B]. These findings demonstrate that relationships exist among different vocal learning phenotypes.

## Species with more-advanced vocal learning abilities are better problem-solvers

We presented all 214 individuals of the 23 bird species with seven cognitive tasks after overnight food deprivation, the duration of which was adjusted to the night lengths and body weights of each individual. The tasks occurred over 6 days in the same sequence, with 5-min intervals between trials or tasks (supplementary methods). The first four were different obstacle-removal tasks of increasing complexity, during which the birds had to figure out how to access a food reward (seeds or worms) by either removing, piercing, or pulling parts of the apparatuses (fig. S1, F to I, and movies S1 to S4). We used the average number of trials required to solve the four problems as our problem-solving measure. Self-control was evaluated using a typical detour-reaching task in which birds had to access a food reward without trying to obtain the food through a transparent barrier (fig. S1J and movies S5 to S6). Associative learning was measured on a standard two-color discrimination task in which birds had to associate a color with a food reward (fig. S1K and movie S7). The rewarded color was switched the next day to assess reversal learning.

**Fig. 2.**
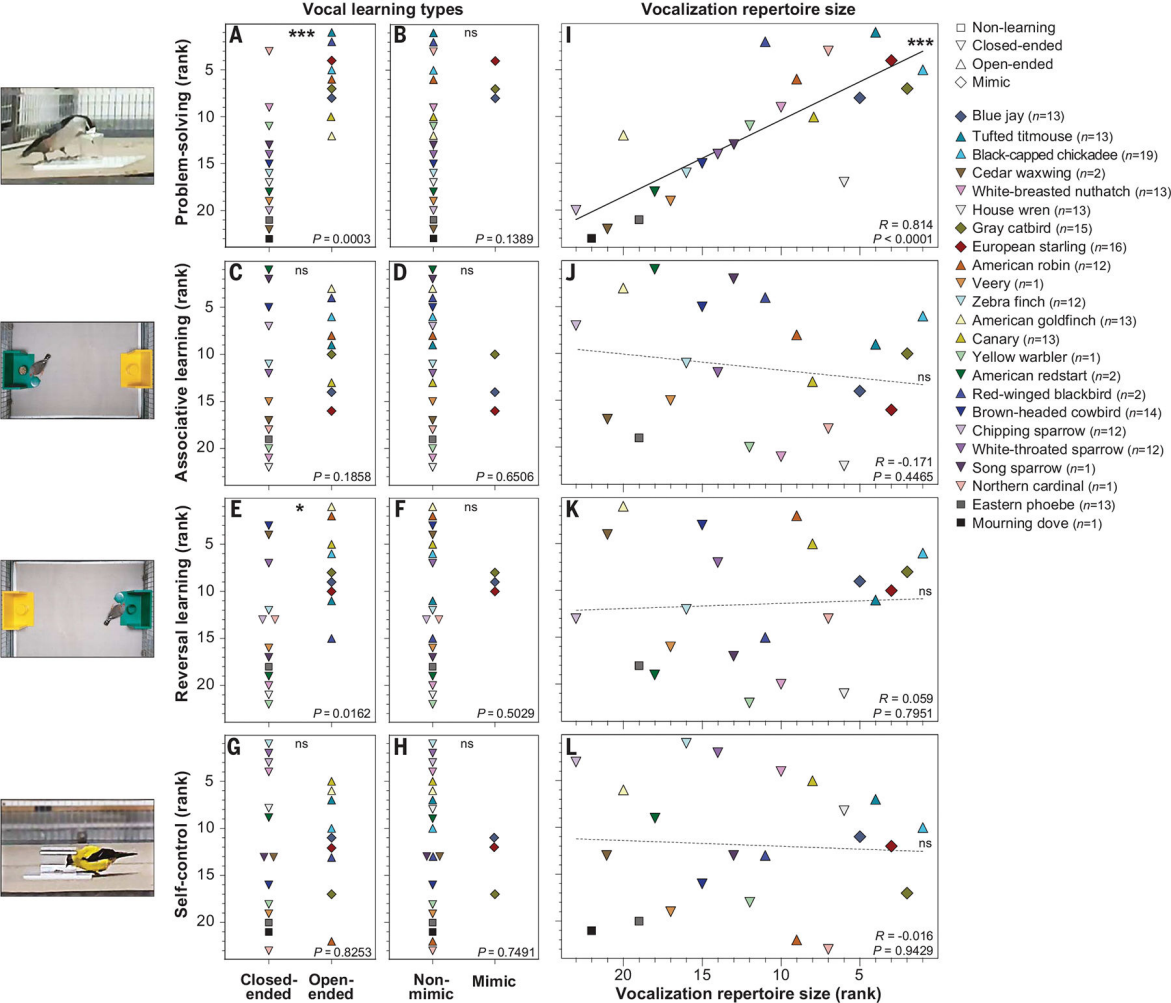
Relationships between cognitive traits and vocal learning features. (**A** to **H**) Four cognitive measures (*y* axes) compared between species grouped by vocal learning features (*x* axes: closed-ended, which includes vocal nonlearning species; open-ended; and nonmimic or mimic). (**I** to **L**) Correlation analyses between the four cognitive behavioral measures (*y* axes) and vocalization repertoire size (*x* axis). Values are ranked means of species performance from all individuals (sample sizes are shown in the legend). *P* values in (A) to (H) are from Wilcoxon tests; *R* and *P* values in (I) to (L) are from Spearman correlations. Regression lines are for illustration purposes to show the significance and direction of relationships. Images to the left are examples of birds in the different tasks, which are snapshots from videos taken by the authors. ***P < 0.001; *P < 0.05; ns, not significant.

Looking at the categorical vocal learning variables, we found that species classified as open-ended vocal learners were significantly better problem-solvers than the other species (Fig. 2A). Species capable of vocal mimicry were at the upper problem-solving performance range, although the difference was not significant (Fig. 2B). There were no significant differences for open-ended or mimicking vocal learning species and their associative learning abilities (Fig. 2, C and D), reversal learning (Fig. 2, E and F), or self-control (Fig. 2, G and H), except a largely overlapping, but significantly better, reversal learning performance in open-ended vocal learners (Fig. 2E). When splitting the vocal learning phenotypes into a more-sensitive multigroup ANOVA analysis, only problem-solving was significantly better for both open-ended vocal learners and mimics (fig. S2, A to D).

Looking at the continuous vocal learning variables, we found a strong and significant relationship, with species having the largest repertoires (songs and calls) being the best problem-solvers (Fig. 2I). We found no significant correlations or even qualitative signs of a correlation between vocalization repertoire size and associative learning, reversal learning, or self-control (Fig. 2, J to L). Considering only songs or calls yielded similar significant relationships with problem-solving, although the associations were weaker (table S3). Excluding vocal nonlearning and domesticated species yielded identical conclusions (fig. S2, F to I). When excluding the eight species with small sample sizes, the correlation between vocalization repertoire size and problem-solving remained (Spearman’s correlation *R* = 0.779; *P* = 0.0006). Together, these results indicate a positive association between vocal learning features and problem-solving among cognitive traits.

## Vocal learning complexity better predicts problem-solving performance

**Fig. 3.**
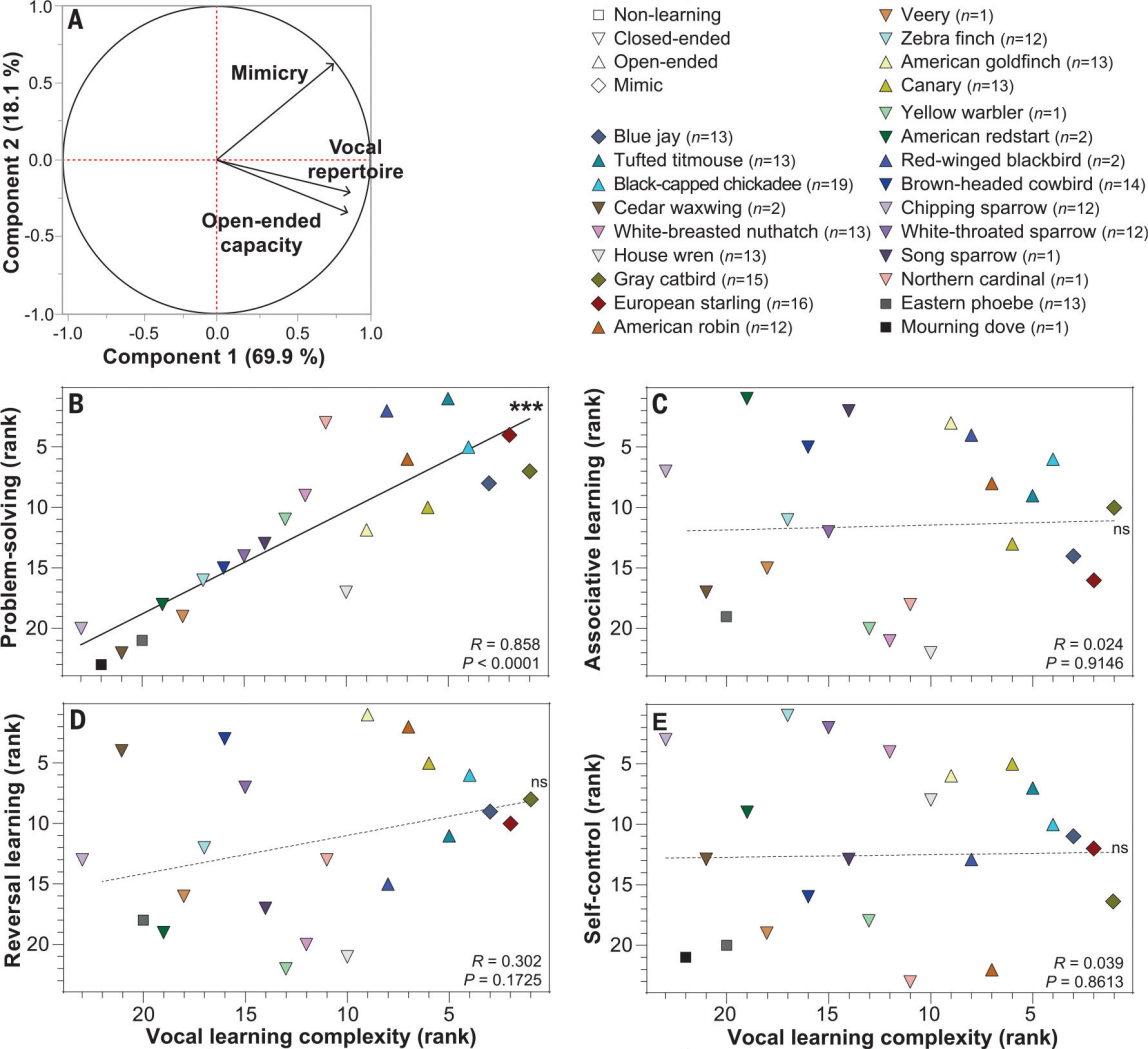
Species that display higher vocal learning complexity are better problem-solvers. (**A**) PCA on open-ended capacity, mimicry capacity, and log of vocalization repertoire (songs and calls). PC1 explains 69.9% of the variance and was used as our index of vocal learning complexity. (**B**) The higher the vocal learning complexity, the better the problem-solving performance among species. (**C** to **E**) Species’ associative learning (C), reversal learning (D), and self-control (E) performances are not associated with vocal learning complexity. Values are ranked means of species performance from all individuals (sample sizes are shown in the legend). *R* and *P* values are from Spearman correlations. ***P < 0.001; ns, not significant.

To obtain an estimate of general vocal learning complexity, we performed a principal components analysis (PCA) with the three vocal learning features (open-endedness, mimicry, and total vocalization repertoire size) and extracted the first principal component (PC1), which explained the majority (69.9%) of the variance in all three measures (Fig. 3A). We found a positive and significant relationship between the vocal learning complexity PC1 and problem-solving across species, which was stronger than with any of the individual vocal learning features (Fig. 3B versus Fig. 2, A to B and I). Vocal learning complexity was unrelated to the other cognitive traits of associative learning, reversal learning, or self-control (Figs. 3, C to E). Again, excluding vocal nonlearning and domesticated species did not change the outcome of the results (fig. S3). When excluding the eight species with small sample sizes, the correlation between vocal learning complexity and problem-solving remained (*R* = 0.846; *P* = 0.0001). These findings strengthen the conclusion of an association between vocal learning abilities and problem-solving across species and provide a means to summarize overall vocal learning complexity in one measure.

## More-advanced vocal learners have bigger brains

**Fig. 4.**
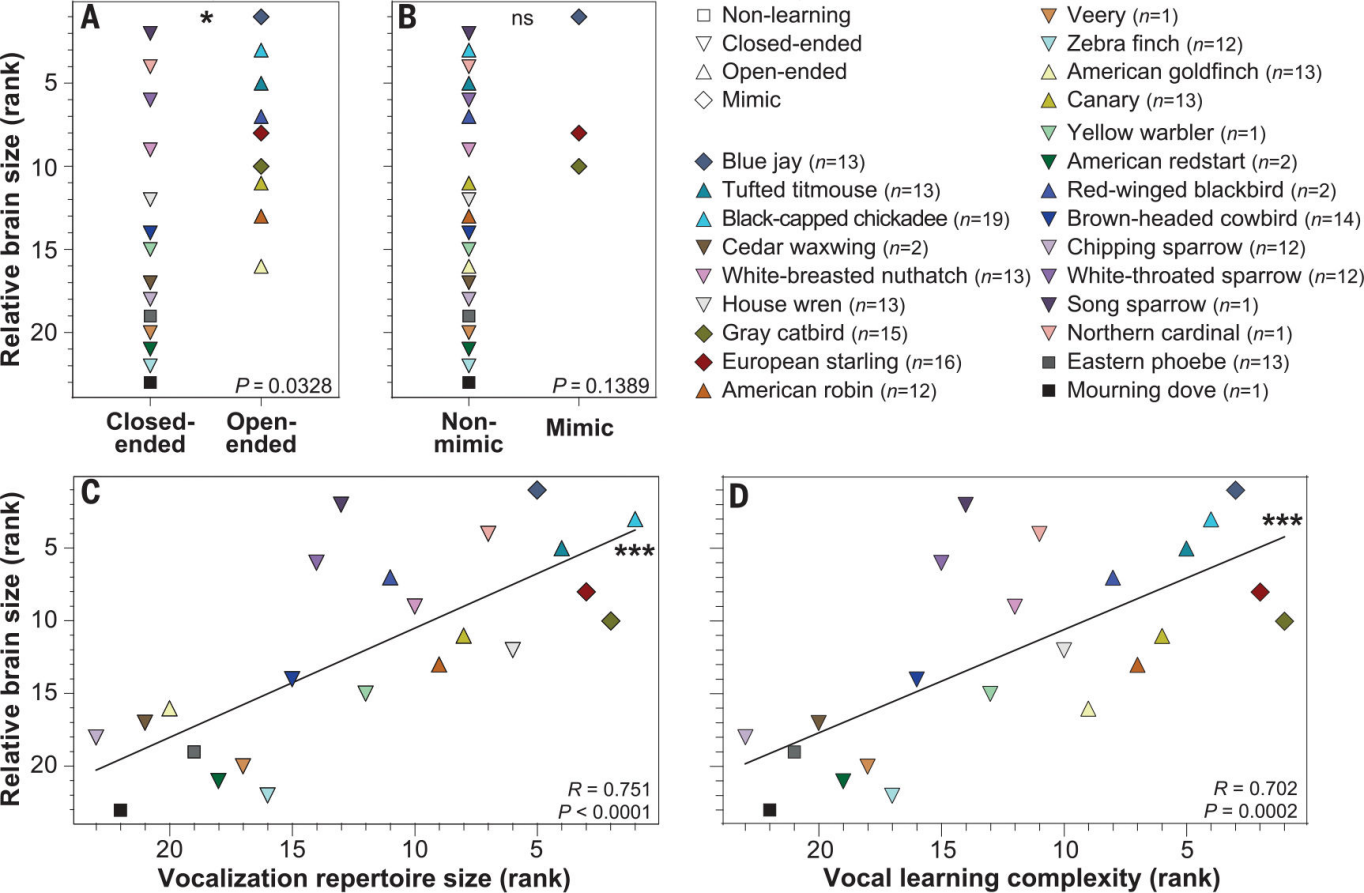
Relationships between relative brain size and vocal learning features. (**A**) Open-ended vocal learning species have significantly larger relative brain sizes than the other species. (**B**) The difference in brain size between mimic and nonmimic species is not significant. (**C**) Vocalization repertoire is significantly associated with relative brain size. (**D**) Species’ vocal learning complexity is also significantly associated with relative brain size. Relative brain sizes are the residuals of brain volumes with body size obtained from (43). *P* values in (A) and (B) are from Wilcoxon tests; *R* and *P* values in (C) and (D) are from Spearman correlations, with vocal learning complexity from PC1 of Fig. 3A. ***P < 0.001; *P < 0.05; ns, not significant.

We sought a biological variable that could explain our findings and examined relative brain size (brain to body size residuals), which, although a coarse measure, was available for all the studied species (21). Previously, relative brain size was found to vary with counts of field innovations (22, 23) and higher neuron numbers (24), and the size of the songbird high vocal center (HVC) vocal learning nucleus was found to vary positively with song repertoire size (25, 26). We found that open-ended vocal learning species had significantly larger relative brain sizes, but there was no significant difference in mimicking species (Fig. 4, A and B). Further, there was a significant positive correlation between vocal repertoire size, as well as overall vocal learning complexity, and relative brain size (Fig. 4, C and D). These relationships again held when excluding vocal nonlearning and domesticated species (figs. S2J and S3E) or species with low sample size (vocal learning complexity *R*_VLC_ = 0.714; *P*_VLC_ = 0.0028). These findings show that vocal learning complexity, problem-solving abilities, and relatively larger brains are all related.

## Vocal learning cognitive relationships are not due to noncognitive factors or phylogeny

We tested whether the relationships that we discovered could (i) also be found when including individual variation, (ii) be explained by noncognitive factors such as personality traits and captive conditions, or (iii) be explained by phylogeny relationships. To address these factors within the same analysis, we used generalized linear mixed models of Markov chain Monte Carlo techniques (MCMCglmm). The models included phylogenetic relationships between species as a random effect, personality traits (shyness, or latency to feed following human disturbance; neophobia, or latency to feed in the presence of a novel object, minus shyness), experimental conditions (capture site, food deprivation period, body weight), and captive status (wild or domesticated) on the whole dataset of the 214 individual values.

Open-endedness, mimicry, vocalization repertoire size, and vocal learning complexity all still significantly predicted problem-solving performance in the MCMCglmm modeling; they were still not associated with associative learning, reversal learning, or self-control, except for reversal learning, which was marginally associated with vocal learning open-endedness (Table 1 and tables S4 to S7). In addition, shyness was negatively associated with problem-solving and positively associated with reversal learning and self-control, neophobia was negatively associated with associative learning, and body weight was negatively associated with self-control but positively associated with reversal learning (Table 1 and tables S4 to S7). Although open-ended vocal learning or mimicry did not predict brain size, repertoire size and vocal learning complexity were significantly associated with relative brain size in the MCMCglmm modeling (Table 1 and tables S5 to S7). Excluding species with small sample sizes yielded similar relationships between vocal learning complexity and all tested cognitive traits (table S8). Thus, MCMCglmm analyses strongly support the existence of a robust relationship between vocal learning abilities, problem-solving, and brain size, even when taking into account individual variation, phylogeny, and other potential confounding covariates.

## Discussion

Our findings suggest a coevolution between vocal learning complexity, problem-solving, and relative brain size. Vocal learning and innovative problem-solving have separately been linked with extinction risk, fitness, and sexual selection (19, 27). Further, both problem-solving and bird songs vary according to habitat differences, for example, urbanization (28, 29). To explain our and these ecological findings, we suggest that a selective factor links these traits within a “vocal learning cognitive complex.” This selective factor could be a genetic component that drives coevolution of vocal learning complexity, problem-solving, and relative brain size.

Previous studies that performed within-species comparisons on vocal learning and other cognitive traits found no or conflicting evidence of relationships [(6, 7, 9, 10, 12), reviewed in (5)]. A major factor as to why we find a clear relationship between vocal learning complexity and problem-solving is likely greater differences across than within species. In addition, some studies trained birds to first solve problems and then measured their capacity to repeat the learned solution, whereas we measured problem-solving on the first trial. We also generated a new measure of vocal learning complexity that did not ignore calls that were assumed to be innate but included calls and songs together. Calls are increasingly recognized as learned in vocal learning species (30, 31).

Our finding of a link between vocal learning complexity and brain size is consistent with the prior finding that two of the three avian vocal learning lineages (songbirds and parrots) have larger relative brain sizes and a higher density of telencephalic neurons compared with vocal nonlearners (32). Devoogd *et al*. (25) found a relationship between song repertoire size and the HVC size across songbird species, but not telencephalon size [confirmed in (26)]. We hypothesize that the relative increase in brain size across species could be due to relative increases in the song system and the adjacent nonvocal motor circuit (33) that is potentially involved in problem-solving. Vocal learners can also synchronize body movements to rhythmic sounds of music (dance), which is thought to be controlled by the surrounding motor circuits (34, 35) and could be another component of a vocal learning cognitive complex. Another brain region to consider is the caudal-lateral nidopallium (NCL), or avian prefrontal cortex, which is involved in complex cognitive processing (36). Regardless of specific brain regions, a higher density of neurons likely provides vocal learners with more brain computational power.

At the molecular level, expression levels of different *N*-methyl-D-aspartate (NMDA) glutamate receptor subunits in song nuclei and nearby brain regions have been associated with both complex vocal learning capacity (37, 38) and problem-solving (39). Thus, this neurotransmitter receptor family is a plausible candidate to partly explain our discovered cognitive relationships.

There has been a long-standing assumption of a link between human spoken language, advanced cognition, and larger brain size relative to other species (40). Our study quantitatively tests such a relationship between species in a vocal learning bird lineage and serves as a model for testing other avian and mammalian lineages. Testing cognitive abilities across more-divergent species might be more challenging because it may require different apparatus designs, whereas this is not the case for comparative genomic studies across species without behavioral testing [e.g., (41)]. For example, some bird lineages have highly divergent beak shapes (e.g., hummingbirds, pelicans) or rely mainly on their feet to manipulate objects (e.g., parrots, birds of prey). Moreover, the vocal repertoires of many nonsongbird species are not as well characterized as those of songbirds. Nevertheless, it would be interesting to see whether the relationships we discovered here exist for other vocal learning species and innate repertoires of vocal nonlearning species. Our results support the continuum hypothesis of vocal learning within a clade (1). More broadly, our discoveries open the door for an unexplored sphere of research on shared neurobiological, molecular, and physiological evolutionary foundations of vocal learning and problem-solving.

## Supplementary Material

Audet et al 2023 SOM 1

Audet et al 2023 SOM 2

Audet et al 2023 SOM 3

science.org/doi/10.1126/science.adh3428

Materials and Methods

Figs. S1 to S3

Tables S1 to S8

References (45-144)

MDAR Reproducibility Checklist

Movies S1 to S7

## Figures and Tables

**Table 1.** Summary of MCMCglmm phylogenetic models. Full, separate models were implemented for vocal learning type [closed-ended (reference category), open-ended, and mimicry]; repertoire size of songs, calls, or both; and vocal learning complexity. All potential covariates were tested first (see tables S4 to S7), and the models with only the significant variables, when applicable (with species, phylogeny, and capture site as random effects), were rerun to obtain unbiased effects of vocal learning. Significant effects are highlighted in bold. All measured behaviors are expressed in logged trials; higher numbers represent lower performance (*n* = 23 species, 214 individuals). Post.mean, mean of the posterior distribution; CI, confidence interval; pMCMC, MCMCglmm *P* value.

Cognitive trait	Vocal learning feature	Post.mean	Lower 95% CI	Upper 95% CI	pMCMC
**Problem-solving**	**Open-endedness**	**−1.151**	**−1.806**	**−0.517**	**0.0020**
	**Mimicry**	**−1.036**	**−2.015**	**−0.056**	**0.0378**
	**Song repertoire**	**−0.174**	**−0.323**	**−0.030**	**0.0217**
	**Call repertoire**	**−0.187**	**−0.342**	**−0.032**	**0.0216**
	**Total vocal repertoire**	**−0.257**	**−0.395**	**−0.118**	**0.0018**
	**Vocal learning complexity**	**−0.090**	**−0.134**	**−0.046**	**0.0012**
Learning	Open-endedness	−0.391	−0.843	0.060	0.0889
	Mimicry	−0.295	−0.890	0.297	0.3065
	Song repertoire	0.024	−0.078	0.126	0.6366
	Call repertoire	−0.038	−0.141	0.068	0.4620
	Total vocal repertoire	−0.009	−0.124	0.106	0.8780
	Vocal learning complexity	−0.016	−0.054	0.021	0.3768
**Reversal learning**	**Open-endedness**	**−0.818**	**−1.497**	**−0.133**	**0.0269**
	Mimicry	−0.607	−1.469	0.272	0.1634
	Song repertoire	0.066	−0.092	0.227	0.4115
	Call repertoire	−0.029	−0.194	0.140	0.7206
	Total vocal repertoire	0.005	−0.173	0.185	0.9675
	Vocal learning complexity	−0.037	−0.093	0.021	0.1978
Self-control	Open-endedness	−0.111	−0.910	0.667	0.7938
	Mimicry	−0.725	−1.928	0.443	0.2026
	Song repertoire	0.003	−0.144	0.148	0.9554
	Call repertoire	−0.009	−0.162	0.147	0.8932
	Totalvocal repertoire	0.002	−0.160	0.169	0.9742
	Vocal learning complexity	−0.023	−0.080	0.034	0.4057
**Relative brain size**	Open-endedness	0.976	−0.390	2.362	0.1571
	Mimicry	1.971	−0.248	4.242	0.0812
	**Song repertoire**	**1.373**	**0.114**	**2.628**	**0.0362**
	**Call repertoire**	**1.702**	**0.392**	**2.989**	**0.0132**
	**Total vocal repertoire**	**2.297**	**1.125**	**3.494**	**0.0013**
	**Vocal learning complexity**	**0.678**	**0.242**	**1.119**	**0.0037**

## Data Availability

The raw dataset and code are available at Dryad (44).
