# Supplementary material for: Songbird species that display more-complex vocal learning are better problem-solvers and have larger brains
Source: Science. Author manuscript; Available in PMC 2026 Mar 26. (PMC13020620; doi:10.1126/science.adh3428)
Supplement: Audet et al 2023 SOM 1 [file NIHMS1967767-supplement-Audet_et_al_2023_SOM_1.pdf]

Science
AAAS

Supplementary Materials for

Songbird species that display more-complex vocal learning are better problem-solvers and have larger brains

Jean-Nicolas Audet, Mélanie Couture, Erich D. Jarvis

Corresponding authors: Jean-Nicolas Audet, jaudet@rockefeller.edu; Erich D. Jarvis, ejarvis@rockefeller.edu

Science **381**, 1170 (2023)
DOI: 10.1126/science.adh3428

The PDF file includes:

Materials and Methods
Figs. S1 to S3
Tables S1 to S8
References

Other Supplementary Material for this manuscript includes the following:

MDAR Reproducibility Checklist
Movies S1 to S7

Materials and Methods

Wild bird captures and acquisition of domestic birds

A total of 189 wild birds of 21 species were caught during 3 years (2018-2020) on the grounds of the Rockefeller University Field Research Center (RUFRC, Millbrook, NY, USA, 41° 46' 3.0" N, 73° 45' 2.5" W) using mist nests deployed on eight sites (4 open fields and 4 forests) in a 30-ha area around the RUFRC main campus (distance between each capture site: 200-500 meters). The mist nets were 36 mm mesh measuring 2.6 m x 12 m (Avinet Research Supplies, Portland, ME, USA). They were installed in the morning and were visited at least every 15 minutes. Upon capture, birds were put in opaque but breathable cotton/poly blend fabric holding bags (Avinet Research Supplies) and immediately brought to the behavior laboratory, where they were weighed, measured, banded, and put into cages (see below). In addition, 12 zebra finches of 9-15 months of age were obtained from the RUFRC domestic colony, and 13 "American Singer" canaries aged 8-16 months were purchased from Stewart's bird farms, Harleysville, PA. After being transferred to the behavior laboratory, the domestic birds were submitted to the same treatment as the wild birds. We initially chose to test only males to reduce potential sex-driven variability in behavior and increase statistical power. However, because catching blue jays and European starlings in sufficient numbers proved challenging in our capture area, we also tested females of those species ($n = 3$ and $n = 4$, respectively). We decided to include the females in the analyses since we did not detect significant differences in their cognitive performance compared to males (for all trait comparisons, all $P > 0.38$). Birds of all species were molecularly sexed (see below). After capture or acquisition from domestic colonies, all birds were measured using the same procedure as in (*28*). The measurements were all performed by the same person (JNA).

Housing conditions

All birds of 23 species were put in custom 81.3 x 55.9 x 68.6 mm aluminum cages with 15.2 x 53.3 mm front doors in an indoor aviary at the RUFRC. The front end of the cages was made of transparent abrasion-resistant polycarbonate to facilitate observations. The birds were visually but not acoustically isolated from each other using opaque corrugated plastic panels that were larger than the cages. All experiments were filmed using a Brio 4K Ultra HD camera (Logitech, Newark, CA, USA). The observations were carried out through the live video feed (in addition to recordings, when required) on a 27'' 4K monitor (Dell, Round Rock, TX, USA) in an adjacent room where the birds could not see or hear the experimenter, who remained the same (JNA) throughout all behavioral tests during the three field seasons. To prevent any stress caused by a change in the birds' circadian rhythm, the light cycle was adjusted to follow the natural light cycle automatically. During the captivity period, all birds were provided with a water bath, refilled daily. During the first 3 days (Friday-Sunday) in the behavior cages, the birds were undisturbed (except for replenishing their food and water once a day) and provided with food *ad libitum*. All birds were provided with sunflower seeds (Ultra Clean sunflowers, Kent Nutrition Group, Muscatine, IA, USA), mealworms (Bug Company, Ham Lake, MN), wax worms (Bug Company), and a species-specific seed mix. The seed mix differed between the species; blue jays, European starlings and red-winged blackbirds received a wild bird mix of seeds, grain, and nuts (Blue Seal Neat Feast, Kent Nutrition Group), canaries a commercial canary seed mix (Blue Seal Colors 'n Chorus canary diet, Kent Nutrition Group), American goldfinches a half-and-half mix of thistle/commercial canary seed mix (Blue Seal Colors 'n Chorus canary diet, Kent Nutrition Group), and all the other

species received a finch seed mix (Blue Seal Colors 'n Chorus Finch Diet, Kent Nutrition Group). If any bird did not eat or drink after one day or looked unhealthy, it was released immediately. All birds, except a few sacrificed for sampling brains for another study, were released at their initial capture site at the end of the captivity period.

Molecular sexing

A total of 16 out of the 23 study species are not sexually dimorphic. Therefore, we determined the sex of all birds (including the sexually dimorphic species, to confirm the visually identified sex) using a standard sex-typing PCR protocol from blood samples (~20 µL) collected by puncturing the brachial vein (*45*, *46*).

Vocal learning features

To obtain estimates of vocal learning characteristics, we performed a literature survey. We first attempted to draw data from existing databases of songbird vocal repertoires (e.g., (*25*, *27*, *47*, *48*)). However, several issues that prevented us from using those databases: 1) not all our species were described in published databases; 2) most of them were outdated (e.g., they often do not include recent data on species' repertoires, or report "infinite/large" or arbitrary "1000" values for species that have large repertoire size, but for which vocalization repertoire estimates are now available; and 3) none of them contained information on call repertoire. Thus, we generated a *de novo* vocal feature database by performing an exhaustive literature survey. We searched Google Scholar for terms "[species]" with "repertoire", "songs", "calls", "vocalizations", "phrases"/"motifs", "open-ended", "imitation" or "mimicry". When data on both wild and captive birds were available, we used wild bird data, as captive birds are likely exposed to fewer vocalization types from which they elaborate their repertoire (see (*49*)).

Vocal repertoire size is typically assessed by counting either the number of song types, or syllable types that an individual of a given species sings on average. Song and syllable repertoires are neither equivalent nor directly comparable. We opted for song-type repertoires because reports of syllable repertoire size were not available for all our study species. When multiple sources were available for a given species, we calculated the average of reported song types sung per individual, from up to 3 of the most representative sources (table S1). Some of our study species sing long, sometimes almost continuous, songs (referred to as *continuous singers*, see, e.g., (*50*); in our study: catbird, starling, robin, and canary). The nature of their songs makes it very difficult to categorize unique song types precisely. For this reason, the reported song repertoire metrics of those species is generally the number of unique "motifs" or "phrases". Because the length of their "phrases/motifs" is often equal to or higher than whole typical songs of non-continuous singing species, this metric is generally assumed to be comparable to song-type repertoires of non-continuous singers (*51*). Importantly, we did not use reported repertoires of other song subdivisions for these, or any other species (e.g., syllable, note).

Although often considered innate, calls in vocal learning species can also be learned (for review, see (*30*, *52*)). Some species have recognized complex call systems, e.g., parids and corvids. Therefore, we included the number of calls in our vocalization repertoire. Our call repertoire size refers to the number of call types that a species can produce (as opposed to the individual repertoire for songs) because this was the only reported metric for the vast majority of species. Consequently,

when the reported calls from multiple citations were claimed as the full species' repertoire, we averaged them, however, when they were specifically reported as additional, non-previously reported calls for the species, we added them. We acknowledge that this value may represent an overestimation of the individual call repertoire since a given individual may not always produce all possible call types of the species. On the other hand, since calls are generally understudied compared to songs, those numbers may underestimate the complete diversity of calls. Taken together, we believe that this metric is an estimation of individual call repertoires. Importantly, we report the same metric for all species; therefore, it is likely not biased in favor of any given species.

We noted that the vocalizations of some species appear to be less studied than others (e.g., American goldfinch, white-breasted nuthatch, see table S1). This low research effort could result in a lower number of measured vocalizations. We tested for this effect, but research effort (from (*53*)) did not significantly correlate with vocalization repertoires (Spearman correlation: R = 0.2441, P = 0.2617, n = 23 species).

The second part of our literature survey aimed at classifying the species as either open-ended or closed-ended vocal learners. Open-ended vocal learning species learn new vocalizations (songs or calls) throughout their life. Closed-ended vocal learning species learn vocalizations at an early stage of life, but their vocalization repertoire more strongly crystallizes near the end of the juvenile development, and their repertoire becomes fixed after this critical period. We categorized species as open-ended vocal learners when we could find evidence showing that a species has open-ended vocal learning capacities; otherwise, they were considered closed-ended (table S1).

The third part of our survey aimed at classifying if our study species were capable of mimicry. Species were categorized as mimics if they were described as capable of commonly imitating extraspecific vocalizations. We excluded rare or anecdotal mentions of mimicry, as including those ambiguous cases would make our classification prone to a certain level of subjectivity. Only widely-accepted mimic species were classified as such (table S1).

Using the above metrics from the literature, we then performed a principal component analysis (PCA, see below for details). The PCA was computed with logged vocalization (song and call) repertoire, open-ended capacity (0: closed-ended, 1: open-ended), and mimicry capacity (0: no report of mimicry found, 1: documented mimicry). The PCA was conducted using the FactoMineR package in R (*54*), a type of Multivariate Exploratory Data Analysis (MEDA) that allows for the grouping of continuous and categorical variables. We extracted the PC1, which explained 69.9 % of the variance, as our vocal learning complexity variable. Excluding the vocal non-learning species (Eastern phoebe and mourning dove) from the analyses that assessed relationships between vocal learning complexity and other traits yielded similar results.

Behavioral tests

General procedure

After the 3-day habituation period, the birds were submitted to our behavioral testing procedure, which lasted for 6 days. Before each testing day, the birds were food-deprived overnight to ensure they were motivated enough to participate in the tasks. Because our species vary considerably in their body mass (~11 g to ~130 g) and night lengths vary throughout the season (8.75 to 14.75

hours), we adjusted the fasting periods for those variables, which are expected to affect the fasting state. We developed the following formula to reach a comparable fasting state for all birds:

$$Deprivation\ time\ (h)\ = 2 \cdot ln\big(Bodyweight\ (g)\big) + 0.2 \cdot Night\ length\ (h) +\ 7$$

For example, for a 10-hour night in June (in NY, USA), an 11 g bird would fast for 13.8 hours while an 85 g bird would fast for 17.9 hours, whereas a 14-hour night in November would require 14.6 and 18.7 hours of fasting for the same birds, respectively. The formula was obtained empirically from pilot experiments assessing the bird's motivation to participate in the behavioral tasks using different fasting times. The birds were closely monitored for any sign of distress from starvation, in which case they were immediately fed. The factor for the night length (0.2) is based on the assumption that animals decrease their metabolism during sleep. Evidence on actual reduction of metabolic rate during sleep in birds is scarce, but studies with different bird species suggest that it would be in the range of ~16% to ~38% reduction (*55–57*), which appears to be similar to mammals (*58*, *59*). We considered a conservative value of 20% reduction in metabolism while sleeping. Each additional hour of sleep would thus require an increase of the fasting time of 0.2 hours to achieve a similar fasting state. This formula was applied to calculate the fasting time for every tested individual; therefore, the fasting period was adjusted to compare species that differ in size but also achieve, as precisely as possible, a similar fasting state for all individuals.

All feeding dishes and behavioral tasks were built in three sizes (small, medium, and large) matched to the species' body mass (body mass for small tasks and dishes: 10-20 g, medium: 21-40 g, large: more than 41 g). To minimize neophobia towards the different tasks, all apparatuses were mounted on the same base (white acrylic plates), with the size also matched to the species' body mass categories. Small plates measured 100 x 100 mm, medium plates measured 125 x 125 mm, and large plates measured 165 x 165 mm.

The feeding dish consisted of a Petri dish (35mm, 60mm, or 100mm, depending on the species' body mass category) glued on square acrylic white plates (fig. S1). The same feeding dish was used throughout the entire captivity period, starting on the first day, to minimize potential neophobia towards the feeding dish and the acrylic plate.

Before beginning the behavioral tests, birds were presented with three types of food (seed mix, mealworm, and softened dog food pellets) simultaneously to determine their preferred food, which was later used in all behavioral tests. No bird chose the dog food, 153 birds chose the mealworm, and 61 chose the seeds. The order for the behavioral tests was fixed for all birds, rather than randomized, to minimize the influence of test order on the bird performance (see (*60*) for a detailed explanation). For the first four days, each day started with personality measurements (see below) followed by cognitive tests. For the remaining two days, only cognitive tests were conducted. The tests started between 7:00 and 12:00, depending on the fasting period calculated for each bird, and stopped no later than 16:00, after which they were allowed to feed *ad libitum* until the start of the next overnight fasting period. There was a 5-minute pause between each behavioral test.

Personality measurements

Shyness was the first measurement taken on days 1 to 4. The feeding dish (fig. S1A) was introduced in the cage, the stopwatch was started, and the observer left the room immediately. The

latency (s) to feed was recorded when the birds first contacted the food. There was no maximum set latency for shyness trials. The birds were allowed to feed for 15 seconds before the food was removed from the cage. This general procedure to measure latency was applied for all subsequent behavioral tasks. Another shyness measurement was taken after the neophobia trial. We used the average of all shyness measurements as our overall shyness variable.

After a 5-minute pause, we evaluated neophobia by presenting a novel object placed next to the feeding dish and recording latency to feed. After another 5-minute pause, the shyness measurement was repeated, and the average of the two shyness latencies was subtracted from the total neophobia latency to obtain "pure" neophobia. The same procedure was repeated on days 2-4, but the novel object was changed on each day (day 1: 4 colored cotton balls, fig. S1B; day 2: 2 stacks of colored *Duplo* blocks, fig. S1C; day 3: 2 Erlenmeyer with colored tapes, fig. S1D; day 4: one inflated purple glove, fig. S1E). We used three sets of neophobia objects with different sizes matched with the body mass category of the species. The maximum allowed latency to feed was set at 2 hours; if the birds did not feed before this limit, they were given a latency of 7201 seconds (which happened 22 times across the 856 neophobia trials). The average "pure" neophobia measures on four days were used as the general neophobia variable.

Cognitive tests

We used a battery of cognitive tasks that assessed problem-solving, associative learning, reversal learning and self-control. Importantly, our problem-solving assessment required the birds to solve problems *by themselves* and did not involve any kind of training before the problem-solving tests (also referred to as "shaping" or "stage-learning" on novel foraging tasks, which are sometimes used in the literature, e.g., (*6*)). Repeating a previously learned solution likely assesses different cognitive processes than solving a new problem for the first time. We used four different problems to increase the precision of the measurement and allow birds to perform in slightly different contexts. Each cognitive task was built in three sizes to match the species' body mass.

On day 1, the birds were presented with the "lid-pulling" problem-solving test, which consisted of a glass flask containing a reward, closed with a loose cork lid that could be removed by either pecking on its sides or grabbing the top wooden handle (fig. S1F and movie S1). The apparatus was first presented opened (i.e., the lid placed beside the glass container) to allow the birds to habituate to the task. It was left inside the cage until the birds ate from it. After a 5-minute pause, the task was presented closed. It was left in the cage for 5 minutes or until the bird solved the problem. If unsuccessful, the birds were given a 5-minute pause, then the next trial started. A maximum of 10 trials was allotted to solve the problem. If the birds did not solve the task in 10 trials, they were considered unsuccessful and were given an arbitrary value of 11 trials. The birds that succeeded within the 10 allowed trials received the task again to confirm their success.

On day 2, the second problem, "lid-flipping", was presented. It consisted of a transparent plastic container loosely closed (closed but not snapped) with a flat plastic lid (fig. S1G and movie S2). The birds could either grab the lid by the side or peck at it from bottom to top to solve the problem. The testing procedure was identical to the one followed for the previous problem.

On day 3, self-control was assessed using a detour-reaching task (fig. S1J and movies S5-S6), following a standard procedure (*39*). The initial training phase was conducted using an opaque

cylinder. The birds had to reach for the reward (seeds or mealworm) to complete a trial, with no time limit. After seven successes, the birds moved on to the testing phase, conducted with an identical but transparent cylinder. The birds had to reach directly for the reward without pecking at any part of the cylinder to succeed. The success criterion was set at seven successful trials in a row, and the maximum number of allotted trials was 50, after which unsuccessful birds were given a score of 51 trials.

On day 4, the birds were given a color-discrimination associative learning task. The apparatus was identical to the lid-flipping task but was painted entirely yellow or green (fig. S1K and movie S7). Before using the lid-flipping apparatus for associative learning, we ensured that all the birds were capable of removing the lids from the containers. The birds that solved the lid-flipping problem on day 1 were given the task for another five "practice" trials until they solved it quickly each time to ensure they mastered it sufficiently. The birds that did not previously solve the task were trained until they mastered it. We used the shaping procedure described in (*39*), but there was no maximum number of trials. In brief, the task was presented opened, half-closed, three-quarter closed, closed upside down, and finally fully closed. Each step had to be succeeded twice before continuing to the next one. Once the birds succeeded, they were given the task for another five "practice" trials. At the end of this phase, all birds could perform the lid-flipping task sufficiently to be tested for associative and reversal learning, except for the mourning dove, which failed despite attempting shaping for more than 50 trials. Therefore, we could not measure associative learning and reversal learning for this species. All birds received the same training on the apparatus; therefore, the procedure likely did not affect their associative learning score. In any case, proficiency in flipping the lids off containers (as long as they are capable of doing it) is not expected to influence the number of trials required to associate a cue with a reward.

The associative learning procedure was similar to the one described in (*60*). First, to habituate the birds to the colored apparatuses, two colored tasks were presented opened on each lateral end of the cage. They were removed after the birds ate from both. Then, to ensure that all birds could open the colored apparatuses, they were given again in switched positions (left to right side and vice versa) but with the lid closed. They were removed when the birds opened both and fed. The birds were then given a color choice trial to account for potential individual or species color preferences. Both tasks were presented closed, again in switched positions, and they were removed after the birds ate from the first opened apparatus, which was considered the preferred color. This color became the non-rewarded color for the associative learning test. For the remaining trials, both colored stages were presented simultaneously, always with alternating positions. They were removed either; 1) immediately if the birds chose the unrewarded color; or 2) after allowing them to eat the worm or the seeds for 10 s if they chose the rewarded color. To be successful, the birds had to choose the rewarded color seven trials in a row. This task had no maximum trial number to ensure that all birds learned the rewarded color using the same success criterion before the subsequent reversal learning test.

On day 5, the reversal learning test was conducted. The same apparatus and procedure for associative learning were followed, but the previously-rewarded color became the non-rewarded color and vice-versa. Trials started in the morning following the associative learning test day, with the rewarded color reversed. The same success criterion of 7 trials in a row was applied. The

maximum allotted trial number was set at 100, after which unsuccessful birds were given a score of 101 trials (which occurred in 6 birds).

On day 6, the birds were given two additional problem-solving tasks. The "lid-piercing" task was made of the same plastic container used for the lid-flipping task, but instead of the plastic lid, the opening was covered with an aluminum foil piece, and secured with a rubber band (fig. S1H and movie S3). The birds had to pierce a hole in the aluminum foil to reach the reward.

Then, on the same day, the "stick-pulling" problem-solving task was given. A plastic container (the same used for lid-flipping) was glued to a wooden stick and inserted into a transparent enclosure (fig. S1I and movie S4). The birds had to pull the stick to move the container out of the plastic enclosure and remove the lid to access the reward. They had a maximum of 10 5-minute trials to solve each problem, after which the unsuccessful birds were given an 11-trial score.

Brain size measurements

Brain and body sizes for each species and were obtained from (*43*). Relative brain sizes were calculated from residuals of a linear model with logged brain volumes as the dependent variable and logged body mass as a fixed independent effect. Brain size data was unavailable for the chipping sparrow (*Spizella passerina*); thus, we used the brain volume of its closest relative, the American tree sparrow (*Spizella arborea*), and scaled it based on their body size difference. Excluding the chipping sparrow from analyses assessing relationships with brain size did not change the outcomes.

Statistical analyses

Statistical analyses were conducted in R version 4.2.2 (*61*). The performance variable used for all cognitive tasks is expressed in trial numbers to the success criterion (see above). Using latencies instead yielded similar results. For problem-solving, the average trial number to solve the four problem-solving tasks was used for each bird.

Analyses of repertoire mean differences between vocal learning types of Fig. 1 and table S2 were computed with phylogenetic ANOVAs using the "aov.phylo" function from the Geiger package in R (*62*). We performed 10000 simulations using the consensus tree from (*42*). For those analyses, the first goal was to determine if open-ended vocal learners had larger repertoires than closed-ended vocal learners, and if vocal learning species capable of mimicry had larger repertoires than vocal learning species not capable of mimicry. Therefore, vocal non-learning species were excluded from those analyses (table S2A). We also performed the same analyses with the vocal non-learning species included (table S2B), which can be interpreted as comparisons of open-ended vocal learning species vs. all other species, and mimic species vs. all the others.

Pairwise comparisons of cognitive performance for each separate vocal learning type (Fig. 2A-H) were computed using Wilcoxon ranked tests ("wilcox.test" function) in R, using the mean trials to succeed for each task in the 23 species. Analyses of associations between cognitive performance (trials) and vocal learning types (fig. S2A-E) were computed through ANOVAs ("aov" function), followed by post-hoc pairwise comparisons with Holm correction ("pairwise.t.test" function) in R. R-squared values were obtained from the corresponding linear models ("lm" function), in which

cognitive traits (trials or residuals of relative brain size) were used (separately for each trait) as the response variable and vocal learning types (categorical variable with three levels: closed, open, and mimic) were used as the grouping variable. We excluded vocal non-learning species from the latter analyses.

Correlations between traits were assessed with Spearman correlations in R, using untransformed species means (n = 23) for each behavioral trait. We plotted the ranked values to illustrate these tested relationships; lower rank values indicate better performance in behavioral tasks, larger repertoire sizes and higher vocal learning complexity.

Multivariate Generalized Linear Mixed Models using Markov chain Monte Carlo techniques (MCMCglmm) with phylogeny correction were computed using the MCMCglmm package in R (*63*). The relationships between each trait of interest were assessed using the whole dataset of 214 individual values. We used all raw logged values; therefore, negative effects of vocalization features and brain size on behavioral measures (trials and latencies) represent positive relationships (e.g., species with larger repertoires require fewer trials to solve a task). A single consensus phylogenetic tree, obtained from (*42*), was used for phylogeny calculations in the models. For models examining measured behavioral traits, we included species, phylogenetic relationships and capture sites as random effects, and potential covariates (fasting time, bodyweight, captive status, shyness, and neophobia) as fixed effects. For relative brain size models, for which individual variation of the trait is unavailable, we computed similar models, but excluding captive condition variables, i.e., capture site, fasting time, and body weight. The following MCMCglmm parameters were used in all models: iterations = 65001; thinning interval = 50; burnin = 15000; and priors for R and G set at V = 1 and nu = 0.002. All models were run 100 times; and the mean of all effects and P-values are reported in tables.

The MCMCglmm results in tables S4-S8 show initial and final model outputs. Initial models include the effect of all tested variables. To confirm that non-significant effects were not influencing the outcomes of the models, we performed stepwise variable selection to remove one-by-one the non-significant effects, until only significant effects remained in the final models. Table 1 summarizes the final models of tables S4-S8.

Additional considerations on choice of study species

We had three overarching considerations for the species and samples sizes chosen:

Within songbird species versus non-songbird species: We aimed at comparing cognitive traits and vocal learning complexity in one main lineage, songbirds, as opposed to within other vocal learning and non-learning lineages. The reasons are that songbirds (oscines) are the most specious vocal learning clade, the most studied, and thus the easiest to find existing vocalization data. This is not the case for parrots and hummingbirds. The relatively homogenous morphologies among songbirds facilitate behavioral testing of cognitive traits on the same apparatus designs. Our behavioral tasks required the birds to perform motor actions, therefore including birds from more phylogenetically distant clades would likely increase morphology variation (e.g., hummingbird species with curved/thin beaks, parrots that rely on their legs to manipulate objects, etc.), which would have complicated the interpretation of our results since the outcomes could have been influenced by morphology rather than cognition. We added the closely-related suboscine species

(Eastern phoebe) and the more distant *Columbidae* (mourning dove) as outgroup comparisons, with sufficiently closer anatomy to handle our cognitive test apparatus. However, we were cautious when interpreting data from those species. We performed most analyses with or without those two outgroup species, and their phylogenetic distance was accounted for in MCMCglmm models.

Sample sizes: The study species can be separated into two subsets according to sample sizes: A) 15 "core" species for which we have a large (≥ 12) sample size, based on their feasibility of capture, assessed at the end of the first year of capture; and B) 8 "opportunistic testing" species that were opportunistically caught and tested while conducting the tests on the other "core" species, but for which reaching a high sample size would have been unrealistic for practical reasons (see legends of Figs. 2-4 for sample size of tested birds for each species). The B subset of species was added to the final dataset to increase the number of species and thus expand the breadth of our conclusions. Since their low sample size might have introduced a level of uncertainty in the cognitive test measurements and could have altered our conclusions, we performed the final MCMCglmm models testing associations between vocal learning complexity and all cognitive traits excluding those 8 species. All the significant effects remained the same (table S8).

Wild versus captive domesticated songbird species: In addition to the 21 wild-caught species, we tested two domesticated species, the canary, and the zebra finch. These two species serve as models for vocal learning research and are by far among the most studied songbirds. We believe that including well-characterized birds raised in the same conditions in our comparative study constituted an opportunity to generate valuable knowledge for both ecology and neuroscience fields. Still, we were cautious when interpreting data from those species, as domestication could have affected relationships between traits. We assessed relationships with or without domesticated species; the conclusions were identical. We also included a "captive status" variable (wild caught or domesticated) in the final MCMCglmm tests (tables S4-8) to take into account this important characteristic, which did not change the overall conclusions.

Supplementary Figures

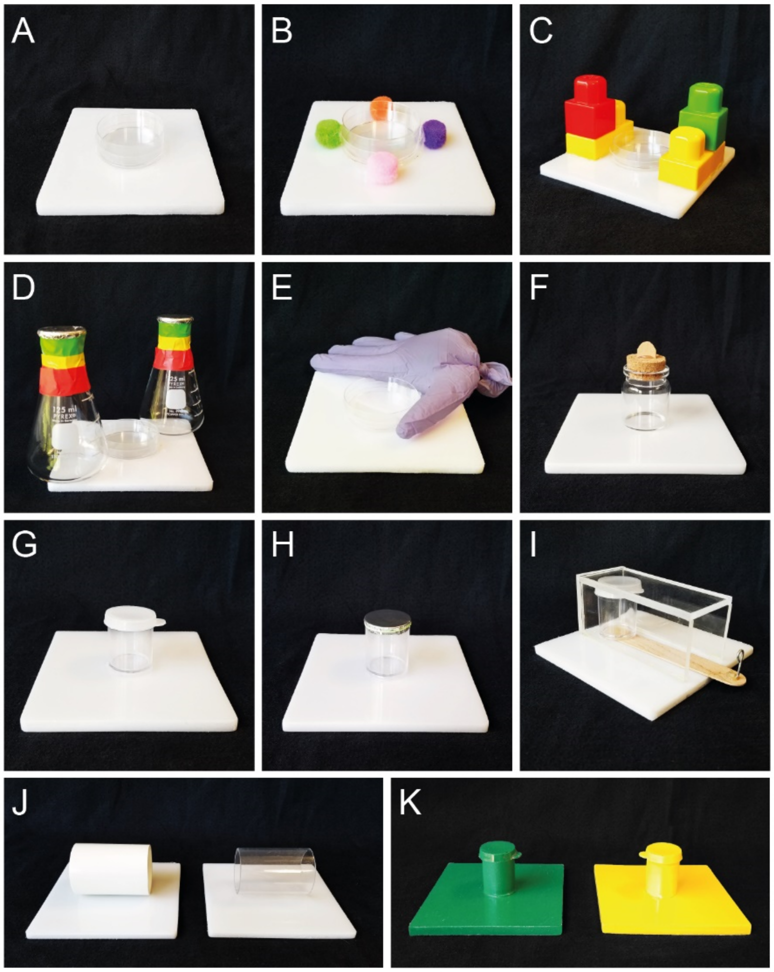

Fig. S1. Behavioral tasks used to assess cognitive abilities and personality traits in 23 avian species. (**A**) Standard feeding dish used throughout the captivity period and used to evaluate shyness. (**B-E**) Novel objects that were used to assess neophobia on days 1 to 4, respectively. (**F**) "Lid-pulling" problem-solving task. (**G**) "Lid-flipping" problem-solving task. (**H**) "Lid-piercing" problem-solving task. (**I**) "Stick-pulling" problem-solving task. (**J**) Detour reaching task. (**K**) Color discrimination learning apparatus used to assess associative and reversal learning. An opaque cylinder was used for the training phase (left) and a transparent cylinder for the testing phase (right). All tasks have been constructed in three sizes matching the body size of the tested birds.

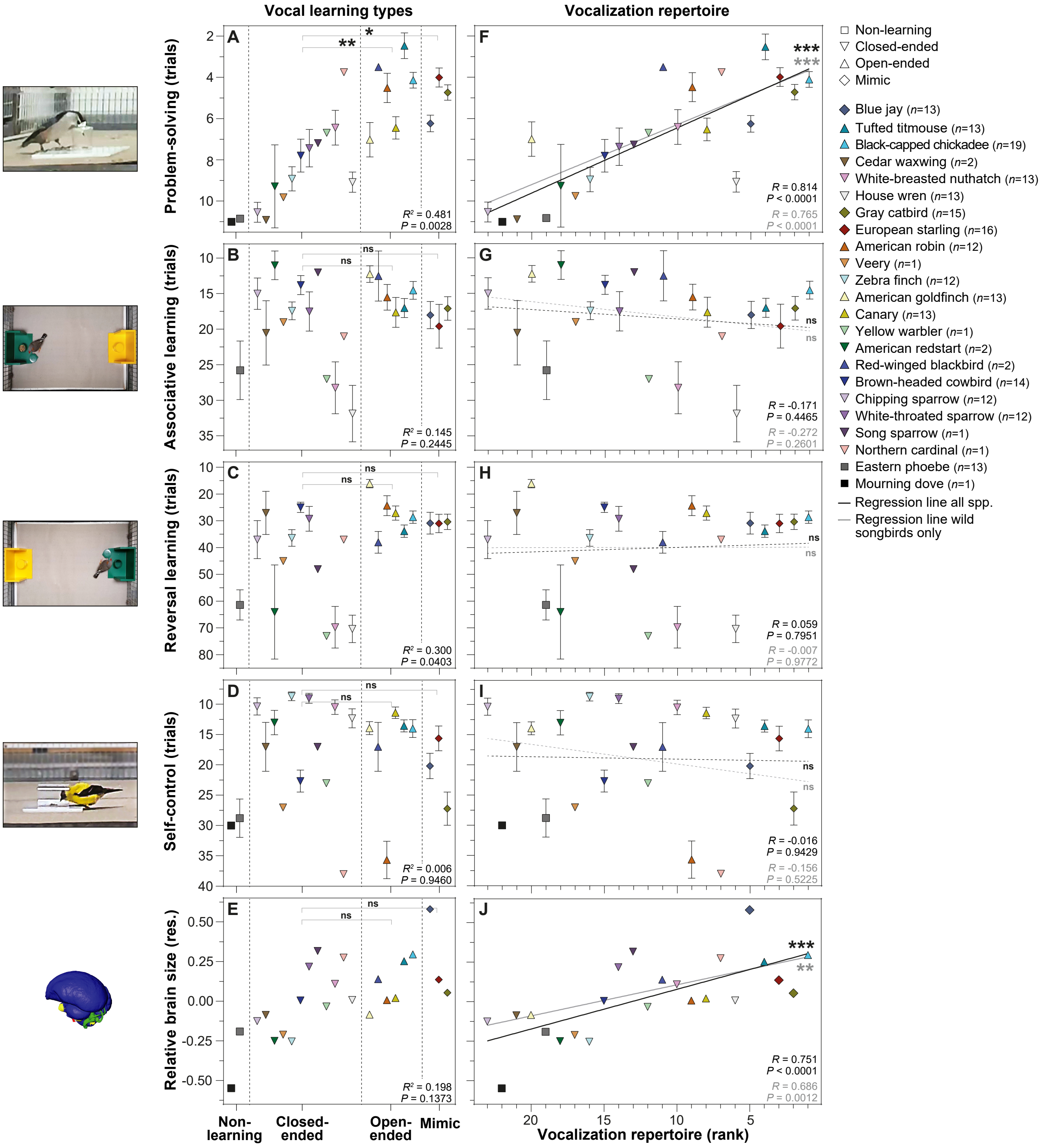

Fig. S2. Relationships between raw cognitive trait values and vocal learning features in each species. Instead of ranks, the average trial number (± SEM) to succeed the behavioral tasks or the residuals of relative brain size per species are shown. (**A**) A one-way ANOVA computed with the three vocal learning types in songbirds (open-endedness, mimicry, and closed-endedness as the reference category) revealed that problem-solving performance (mean number of trials to solve 4 problems) is significantly associated with species' vocal learning types (Post-hoc pairwise comparisons with Holm correction: $P_{open\text{-}endedness}$ = 0.005, $P_{mimicry}$ = 0.034). (**B**) Associative

learning performance does not differ between species of different vocal learning phenotypes (Pairwise comparisons with Holm correction: $P_{open\text{-}ended} = 0.290$, $P_{mimicry} = 0.780$). (**C**) Overall, reversal learning significantly differs between vocal learning phenotypes (Pairwise comparisons with Holm correction: $P_{open\text{-}ended} = 0.059$, $P_{mimicry} = 0.214$). (**D**) Self-control is not significantly associated with vocal learning phenotypes (Pairwise comparisons with Holm correction: $P_{open\text{-}ended} = 1.000$, $P_{mimicry} = 1.000$). (**E**) Relative brain size is not significantly associated with vocal learning types (Pairwise comparisons with Holm correction: $P_{open\text{-}ended} = 0.580$, $P_{mimicry} = 0.170$). (**F**) Species' problem-solving performance is positively and significantly associated with their vocalization repertoire size (number of unique songs and calls). (**G**) Associative learning, (**H**) reversal learning, and (**I**) self-control performances are not associated with species' vocalization repertoire size. (**J**) Species with larger relative brain sizes have significantly larger vocalization repertoires. For panels A-E, statistics were obtained by computing ANOVAs and for panels F-J, Spearman correlations (black regression lines: using all 23 species; gray regression lines: using 19 species of wild songbirds). Relative brain sizes are the residuals of brain volumes with body size obtained from **(*43*)**. Non-learning vocal species are included in graphs of A-E for information but were not included in ANOVA analyses. Regression lines are for illustration purposes, to show significance of relationships.

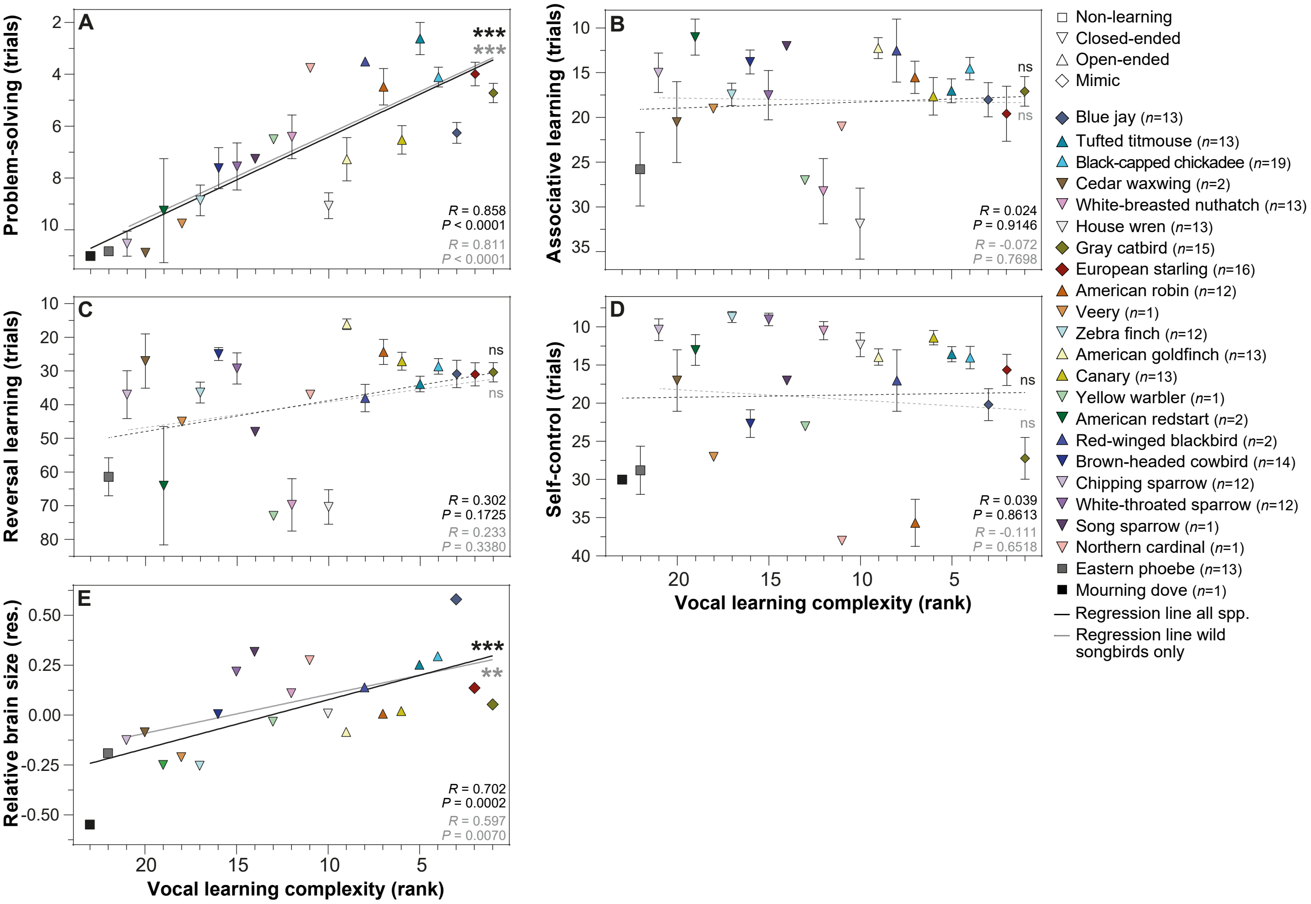

Fig. S3. Relationships between raw cognitive trait values and vocal learning complexity in each species. Instead of ranks, the average trial number (± SEM) to succeed the behavioral tasks or the residuals of relative brain size per species are shown. (A) Species with better problem-solving performance display a significantly higher vocal learning complexity (PC1 from Fig. 3A). (B) Species' associative learning, (C) reversal learning, and (D) self-control performances are not associated with vocal learning complexity. (E) Species' vocal learning complexity is significantly and positively associated with their relative brain size. Statistics were obtained from Spearman correlations (black regression lines: using all 23 species; gray regression lines: using 19 species of wild songbirds). Relative brain sizes are the residuals of brain volumes with body size obtained from **(*23*)**. Regression lines are for illustration purposes, to show significance of relationships.

Table S1. Vocal learning complexity features: vocalization repertoires, open-ended vocal learning and mimicry capacities, with supporting references.

Species name	Repertoire			Open/Closed-ended	Mimicry	Song references	Call references
	Songs	Calls	Total				
American goldfinch *Spinus tristis*	2.00	5.00	7.00	Open (*64*)	No	2 (*65*)	5 (*65*)
American redstart *Setophaga ruticilla*	4.35	4.00	8.35	Closed	No	4.4 (*66*); 4.3 (*67*)	3 (*68*) +1 (*69*)
American robin *Turdus migratorius*	12.99	7.67	20.66	Open (*70*)	No	10 (*71*); 12.47 (*51*); 12-21 (*72*)	10 (*71*); 3 (*51*); 10 (*73*)
Black-capped chickadee *Poecile atricapillus*	3.50	374.00	377.50	Open (*74*)	No	4 (*75*); 3 (*76*)	12 (*77*) +362 (*78*)
Blue Jay *Cyanocitta cristata*	3.00	45.00	48.00	Open (*79*)	Yes (*79*)	1 (*80*); 2 (*81*); 6 (*82*)	41 (*80*) +4 (*81*)
Brown-headed cowbird *Molothrus ater*	5.09	4.00	9.09	Closed	No	5.2 (*83*); 5.46 (*84*); 4.6 (*85*)	(*86*, *87*)
Canary *Serinus canaria*	19.00	2.00	21.00	Open (*88*)	No	8-16 (*89*); 12-24 (*90*); 27 (*91*)	(*92*)
Cedar waxwing *Bombycilla cedrorum*	0.00	6.00	6.00	Closed	No	(*93*)	(*93*)
Chipping sparrow *Spizella passerina*	1.00	3.00	4.00	Closed	No	(*94*, *95*)	(*96*, *97*)
Eastern phoebe *Sayornis phoebe*	2.00	6.00	8.00	Non-learner (*98*)	No	(*98*, *99*)	(*99*, *100*)
European starling *Sturnus vulgaris*	66.03	10.00	76.03	Open (*49*)	Yes (*101*)	71 (*102*); 70 (*103*); 57.1 (*49*)	(*104*)
Gray catbird *Dumetella carolinensis*	146.00	3.00	149.00	Open (*105*)	Yes (*105*)	117 (*72*); 175 (*106*)	(*105*)
House wren *Troglodytes aedon*	30.00	3.00	33.00	Closed	No	24 (*107*); 36 (*108*)	2 (*109*) +1 (*107*)

Mourning dove<br>*Zenaida macroura*	1.00	4.50	5.50	Non-learner (*110*)	No	(*111*)	4 (*112*); 5 (*113*)
Northern cardinal<br>*Cardinalis cardinalis*	9.93	16.00	25.93	Closed	No	9.5 (*114*); 10 (*115*); 10.3 (*116*)	(*117*)
Red-winged blackbird<br>*Agelaius phoeniceus*	5.60	11.00	16.60	Open (*118*)	No	5.2 (*119*); 6 (*120*)	5 (*121*) +7 (*122*); 10 (*123*)
Song sparrow<br>*Melospiza melodia*	8.88	5.00	13.88	Closed	No	5-11 (*124*); 9.2-10.3 (*125*)	(*126*)
Tufted titmouse<br>*Baeolophus bicolor*	11.55	52.00	63.55	Open (*127*)	No	13.7 (*128*); 9.4 (*129*)	18 (*130*) +34 (*131*)
Veery<br>*Catharus fuscescens*	2.83	6.00	8.83	Closed	No	2 (*72*); 3 (*132*); 2-5 (*133*)	(*134*)
White-breasted nuthatch<br>*Sitta carolinensis*	3.00	15.00	18.00	Closed	No	2 (*135*); 4 (*136*)	(*135*)
White-throated sparrow<br>*Zonotrichia albicollis*	3.00	8.00	11.00	Closed	No	2 (*137*); 4 (*138*)	(*139*)
Yellow warbler<br>*Setophaga petechia*	10.55	5.00	15.55	Closed	No	12.3 (*140*); 8.83 (*141*)	(*142*)
Zebra finch<br>*Taeniopygia guttata*	1.00	8.00	9.00	Closed	No	(*89*, *143*)	(*143*, *144*)

Table S2. Results of phylogenetic ANOVA tests comparing repertoires between vocal learning types. (A) Comparisons of repertoires (songs only, or songs and calls) between open-ended and closed-ended songbirds, and between mimic and non-mimic species, taking into account phylogeny. When comparing song repertoires, we considered open-ended the species for which only the song system is known to be open-ended. When comparing song and call repertoire, we considered open-ended the species for which the song or the call system is open-ended (n = 21 species). B) The same comparisons were made, but using the dataset of all species, considering the two vocal non-learning species as closed-ended and non-mimic. Significant associations are highlighted in bold (n = 23 species).

A) Songbirds

Vocal learning feature	Repertoire	Df	Sum Sq	Mean Sq	F Value	P	P (phylo)
**Open-endedness**	**Songs**	1	1.5211	1.52115	7.6336	**0.0124**	**0.0192**
	Residuals	*19*	*3.786*	*0.199*			
**Open-endedness**	**Songs + Calls**	1	1.7665	1.76654	11.171	**0.0034**	**0.0094**
	Residuals	*19*	*3.005*	*0.158*			
Mimicry	Songs	1	1.4655	1.4655	7.2481	**0.0144**	0.0747
	Residuals	*19*	*3.842*	*0.202*			
Mimicry	Songs + Calls	1	1.261	1.261	6.8255	**0.0171**	0.0771
	Residuals	*19*	*3.510*	*0.185*			

B) All species

Vocal learning feature	Repertoire	Df	Sum Sq	Mean Sq	F Value	P	P (phylo)
**Open-endedness**	**Songs**	1	1.7829	1.78291	9.387	**0.0059**	**0.0039**
	Residuals	*21*	*3.989*	*0.190*			
**Open-endedness**	**Songs + Calls**	1	2.1071	2.10714	14.202	**0.0011**	**0.0031**
	Residuals	*21*	*3.116*	*0.148*			
**Mimicry**	**Songs**	1	1.6435	1.64349	8.3607	**0.0087**	**0.0309**
	Residuals	*21*	*4.128*	*0.197*			
**Mimicry**	**Songs + Calls**	1	1.4257	1.42569	7.8847	**0.0105**	**0.0381**
	Residuals	*21*	*3.797*	*0.181*			

Table S3. Spearman ranked correlations between species' song or call repertoires and measured cognitive traits. All measured behaviors (except brain size) are expressed in trials, therefore higher numbers represent lower performance (e.g., negative relationships with repertoire means larger repertoires are associated with better performance). Significant correlations are highlighted in bold (n = 23 species).

Cognitive trait	Repertoire	Rho	p-value
**Problem-solving**	**Songs only**	-0.6512	**0.0008**
**Problem-solving**	**Calls only**	-0.5657	**0.0049**
Learning	Songs only	0.0662	0.7698
Learning	Calls only	0.1100	0.6261
Reversal learning	Songs only	0.0501	0.8249
Reversal learning	Calls only	-0.0476	0.8333
Self-control	Songs only	0.1774	0.4179
Self-control	Calls only	0.0010	0.9964
**Relative brain size**	**Songs only**	0.4957	**0.0162**
**Relative brain size**	**Calls only**	0.5250	**0.0101**

Table S4. MCMCglmm phylogenetic models assessing relationships between all measured behaviors, brain size, and vocal learning types. Vocal learning type (open-ended vocal learning, mimicry, and closed-ended [reference]), personality traits and experimental conditions (except for brain size) were tested as categorical fixed effects, and species, phylogeny and capture site were included as random effects. All variables were included in initial models (above dashed line), and then they were run again with only vocal learning type and the significant covariate(s) when applicable (below dashed line). All measured behaviors (but not brain size) are expressed in logged trials; therefore, higher numbers represent lower performance. Significant effects are highlighted in bold and random effects are in italics (n = 23 species, 214 individuals).

Dependent variable	Independent variable	Post mean	$l_{95\%}$ CI	$u_{95\%}$ CI	Eff samp	pMCMC
Problem-solving	(Intercept)	1.906	-4.097	7.931	947	0.5371
	**VLT: Open-endedness**	**-1.151**	**-1.806**	**-0.517**	**717**	**0.0020**
	**VLT: Mimicry**	**-1.036**	**-2.015**	**-0.056**	**904**	**0.0378**
	**Shyness**	**0.381**	**0.136**	**0.628**	**718**	**0.0029**
	Neophobia	0.050	-0.206	0.305	993	0.7038
	Bodyweight	-0.712	-1.867	0.455	851	0.2237
	Fasting period	1.257	-4.126	6.694	996	0.6497
	Captive status: wild	-0.204	-1.137	0.743	967	0.6477
	Phylogeny	*0.636*	*0.000*	*1.583*	*450*	
	Species	*0.085*	*0.000*	*0.350*	*333*	
	Capture site	*0.039*	*0.000*	*0.159*	*946*	
	(Intercept)	2.212	1.131	3.482	552	0.0013
	**VLT: Open-endedness**	**-1.181**	**-1.790**	**-0.577**	**698**	**0.0015**
	**VLT: Mimicry**	**-1.271**	**-2.119**	**-0.429**	**832**	**0.0054**
	**Shyness**	**0.360**	**0.123**	**0.599**	**759**	**0.0035**
	Phylogeny	*0.563*	*0.000*	*1.434*	*396*	
	Species	*0.093*	*0.000*	*0.342*	*319*	
	Capture site	*0.031*	*0.000*	*0.123*	*941*	
Associative learning	(Intercept)	-2.488	-8.512	3.617	995	0.4204
	VLT: Open-endedness	-0.400	-0.889	0.091	877	0.1079
	VLT: Mimicry	-0.146	-0.878	0.581	1002	0.6791
	Shyness	-0.084	-0.343	0.177	988	0.5258
	Neophobia	0.258	-0.012	0.530	776	0.0627
	Bodyweight	-0.829	-1.856	0.182	904	0.1056
	Fasting period	4.011	-1.646	9.683	931	0.1646
	Captive status: wild	0.255	-0.462	0.983	965	0.4744
	Phylogeny	*0.283*	*0.001*	*0.702*	*535*	
	Species	*0.036*	*0.000*	*0.154*	*483*	
	Capture site	*0.013*	*0.000*	*0.050*	*940*	
	(Intercept)	1.254	0.452	2.077	470	0.0060
	VLT: Open-endedness	-0.391	-0.843	0.060	872	0.0889

	VLT: Mimicry	-0.295	-0.890	0.297	954	0.3065
	**Neophobia**	**0.277**	**0.030**	**0.529**	**636**	**0.0281**
	Phylogeny	*0.253*	*0.001*	*0.592*	*556*	
	Species	*0.025*	*0.000*	*0.108*	*502*	
	Capture site	*0.011*	*0.000*	*0.039*	*951*	
Reversal learning	(Intercept)	1.879	-4.236	8.067	980	0.5534
	VLT: Open-endedness	-0.747	-1.445	-0.015	817	0.0524
	VLT: Mimicry	-0.194	-1.208	0.834	984	0.6812
	**Shyness**	**-0.412**	**-0.666**	**-0.160**	**928**	**0.0020**
	Neophobia	0.006	-0.255	0.265	1007	0.9449
	Bodyweight	-1.240	-2.521	0.023	998	0.0521
	Fasting period	2.437	-3.142	8.018	987	0.3985
	Captive status: wild	0.422	-0.681	1.529	671	0.4486
	Phylogeny	*0.396*	*0.000*	*1.471*	*313*	
	Species	*0.220*	*0.000*	*0.566*	*364*	
	Capture site	*0.026*	*0.000*	*0.093*	*959*	
	(Intercept)	3.518	2.449	4.691	757	0.0010
	**VLT: Open-endedness**	**-0.818**	**-1.497**	**-0.133**	**854**	**0.0269**
	VLT: Mimicry	-0.607	-1.469	0.272	981	0.1634
	**Shyness**	**-0.396**	**-0.646**	**-0.151**	**910**	**0.0023**
	Phylogeny	*0.434*	*0.000*	*1.427*	*334*	
	Species	*0.188*	*0.000*	*0.509*	*337*	
	Capture site	*0.026*	*0.000*	*0.092*	*961*	
Detour reaching	(Intercept)	3.683	-2.701	10.167	907	0.2630
	VLT: Open-endedness	0.059	-0.639	0.738	983	0.8301
	VLT: Mimicry	-0.576	-1.636	0.430	941	0.2472
	**Shyness**	**-0.544**	**-0.811**	**-0.281**	**654**	**0.0010**
	Neophobia	-0.057	-0.325	0.210	974	0.6781
	**Bodyweight**	**2.128**	**0.908**	**3.392**	**667**	**0.0013**
	Fasting period	-4.469	-10.207	1.305	919	0.1258
	Captive status: wild	0.627	-0.359	1.627	967	0.1986
	Phylogeny	*0.448*	*0.000*	*1.561*	*319*	
	Species	*0.210*	*0.000*	*0.619*	*318*	
	Capture site	*0.010*	*0.000*	*0.038*	*961*	
	(Intercept)	-0.719	-2.745	1.314	796	0.4559
	VLT: Open-endedness	-0.111	-0.910	0.667	968	0.7938
	VLT: Mimicry	-0.725	-1.928	0.443	944	0.2026
	**Shyness**	**-0.519**	**-0.786**	**-0.259**	**680**	**0.0010**
	**Bodyweight**	**1.885**	**0.703**	**3.134**	**691**	**0.0021**
	Phylogeny	*0.534*	*0.000*	*2.131*	*246*	
	Species	*0.346*	*0.000*	*0.871*	*295*	
	Capture site	*0.012*	*0.000*	*0.046*	*951*	
Relative brain size	(Intercept)	0.011	-3.333	3.219	626	0.7828
	VLT: Open-endedness	1.054	-0.381	2.494	894	0.1414
	VLT: Mimicry	1.928	-0.354	4.209	813	0.0905
	Shyness	0.000	-0.027	0.027	480	0.9607

Neophobia	0.000	-0.030	0.030	448	0.9645
Captive status: wild	0.655	-1.550	2.855	626	0.5558
Phylogeny	*4.513*	*0.193*	*11.759*	*148*	
Species	*1.011*	*0.000*	*2.786*	*179*	
(Intercept)	0.722	-2.266	3.202	330	0.4943
VLT: Open-endedness	0.976	-0.390	2.362	922	0.1571
VLT: Mimicry	1.971	-0.248	4.242	811	0.0812
Phylogeny	*4.527*	*0.160*	*11.309*	*177*	
Species	*0.865*	*0.000*	*2.538*	*177*	

Table S5. MCMCglmm phylogenetic models assessing relationships between all measured behaviors, brain size and vocalization repertoire. Vocalization repertoire size, personality traits and experimental conditions (except for brain size) were included as fixed effects, and species, phylogeny and capture site as random effects. All variables were included in initial models (above dashed line), and then they were run again with only vocalization repertoire size and the significant covariate(s) when applicable (below dashed line). All measured behaviors (but not brain size) are expressed in logged trials. Significant effects are highlighted in bold and random effects are in italics (n = 23 species, 214 individuals).

Dependent variable	Independent variable	Post mean	$l_{95\%}$ CI	$u_{95\%}$ CI	Eff samp	pMCMC
**Problem-solving**	(Intercept)	1.375	-0.182	2.930	1001	0.0890
	**Vocalization repertoire**	**-0.232**	**-0.368**	**-0.096**	**1001**	**0.0027**
	**Shyness**	**0.125**	**0.062**	**0.189**	**1021**	**0.0012**
	Neophobia	0.029	-0.036	0.095	1022	0.3837
	Bodyweight	-0.210	-0.477	0.052	1008	0.1164
	Fasting period	-0.056	-1.487	1.386	998	0.9377
	Captive status: wild	-0.061	-0.337	0.224	1006	0.6411
	Phylogeny	*0.018*	*0.000*	*0.055*	*751*	
	Species	*0.009*	*0.000*	*0.022*	*870*	
	Capture site	*0.008*	*0.000*	*0.027*	*974*	
	(Intercept)	0.993	0.732	1.253	1005	0.0010
	**Vocalization repertoire**	**-0.257**	**-0.395**	**-0.118**	**993**	**0.0018**
	**Shyness**	**0.114**	**0.052**	**0.176**	**1023**	**0.0013**
	Phylogeny	*0.014*	*0.000*	*0.050*	*698*	
	Species	*0.013*	*0.001*	*0.027*	*920*	
	Capture site	*0.006*	*0.000*	*0.020*	*988*	
Associative learning	(Intercept)	0.346	-1.216	1.906	1018	0.6646
	Vocalization repertoire	-0.005	-0.127	0.119	1031	0.9317
	Shyness	-0.015	-0.081	0.049	1014	0.6474
	**Neophobia**	**0.076**	**0.008**	**0.142**	**1007**	**0.0279**
	Bodyweight	-0.213	-0.471	0.039	1025	0.0993
	Fasting period	0.946	-0.511	2.380	1024	0.2029
	Captive status: wild	0.067	-0.148	0.286	992	0.5270
	Phylogeny	0.019	0.000	0.048	922	
	Species	0.004	0.000	0.013	929	
	Capture site	*0.003*	*0.000*	*0.008*	*969*	
	(Intercept)	1.215	0.965	1.471	1010	0.0010
	Vocalization repertoire	-0.009	-0.124	0.106	1001	0.8780
	**Neophobia**	**0.080**	**0.017**	**0.142**	**998**	**0.0148**
	Phylogeny	*0.017*	*0.000*	*0.040*	*922*	
	Species	*0.004*	*0.000*	*0.011*	*941*	
	Capture site	*0.002*	*0.000*	*0.007*	*986*	

Reversal learning	(Intercept)	1.527	0.063	3.013	1014	0.0450
	Vocalization repertoire	-0.003	-0.186	0.183	986	0.9484
	**Shyness**	**-0.095**	**-0.154**	**-0.037**	**1000**	**0.0021**
	Neophobia	0.009	-0.052	0.069	1023	0.7838
	**Bodyweight**	**-0.335**	**-0.647**	**-0.029**	**1038**	**0.0327**
	Fasting period	0.462	-0.845	1.784	1025	0.4940
	Captive status: wild	0.107	-0.200	0.418	897	0.4918
	Phylogeny	*0.048*	*0.000*	*0.127*	*636*	
	Species	*0.013*	*0.000*	*0.038*	*627*	
	Capture site	*0.003*	*0.000*	*0.009*	*962*	
	(Intercept)	2.075	1.555	2.603	1016	0.0010
	Vocalization repertoire	0.005	-0.173	0.185	991	0.9675
	**Shyness**	**-0.095**	**-0.153**	**-0.038**	**1004**	**0.0020**
	**Bodyweight**	**-0.263**	**-0.515**	**-0.017**	**1014**	**0.0350**
	Phylogeny	*0.054*	*0.000*	*0.128*	*725*	
	Species	*0.010*	*0.000*	*0.032*	*652*	
	Capture site	*0.003*	*0.000*	*0.009*	*987*	
Self-control	(Intercept)	1.655	0.201	3.105	993	0.0276
	Vocalization repertoire	0.011	-0.145	0.168	960	0.8976
	**Shyness**	-0.115	-0.172	-0.057	1021	**0.0011**
	Neophobia	-0.008	-0.068	0.053	1016	0.7992
	**Bodyweight**	0.419	0.156	0.683	1023	**0.0025**
	Fasting period	-0.853	-2.164	0.454	1008	0.2049
	Captive status: wild	0.131	-0.111	0.377	1004	0.2777
	Phylogeny	*0.028*	*0.000*	*0.081*	*710*	
	Species	*0.010*	*0.000*	*0.028*	*748*	
	Capture site	*0.002*	*0.000*	*0.006*	*987*	
	(Intercept)	0.857	0.398	1.322	1020	0.0028
	Vocalization repertoire	0.002	-0.160	0.169	968	0.9742
	**Shyness**	**-0.109**	**-0.166**	**-0.053**	**1028**	**0.0011**
	**Bodyweight**	**0.349**	**0.118**	**0.590**	**1003**	**0.0026**
	Phylogeny	*0.032*	*0.000*	*0.096*	*633*	
	Species	*0.013*	*0.000*	*0.035*	*712*	
	Capture site	*0.002*	*0.000*	*0.007*	*991*	
**Relative brain size**	(Intercept)	-2.311	-5.575	0.782	392	0.1620
	**Vocalization repertoire**	2.355	1.133	3.599	467	**0.0012**
	Shyness	0.000	-0.027	0.027	501	0.9649
	Neophobia	0.000	-0.030	0.030	453	0.9623
	Captive status: wild	0.710	-0.900	2.285	926	0.3614
	Phylogeny	*3.592*	*0.224*	*7.575*	*221*	
	Species	*0.382*	*0.000*	*1.417*	*234*	
	(Intercept)	-1.749	-4.545	0.901	257	0.2465
	**Vocalization repertoire**	**2.297**	**1.125**	**3.494**	**509**	**0.0013**
	Phylogeny	*3.738*	*0.336*	*7.436*	*224*	
	Species	*0.288*	*0.000*	*1.187*	*250*	

Table S6. MCMCglmm phylogenetic models assessing relationships between all measured behaviors, brain size and (A) song or (B) call repertoire. Song or call repertoire size, personality traits and experimental conditions (except for brain size) were included as fixed effects, and species, phylogeny and capture site as random effects. All variables were included in initial models (above dashed line), and then they were run with only song or call repertoire size and the significant covariate(s) when applicable (below dashed line). All measured behaviors (but not brain size) are expressed in logged trials; therefore, higher numbers represent lower performance. Significant effects are highlighted in bold and random effects are in italics (n = 23 species, 214 individuals).

A) Song repertoire

Dependent variable	Independent variable	Post mean	$l_{95\%}$ CI	$u_{95\%}$ CI	Eff samp	pMCMC
**Problem-solving**	(Intercept)	1.396	-0.219	3.009	1009	0.0951
	**Song repertoire**	**-0.149**	**-0.296**	**-0.005**	**993**	**0.0440**
	**Shyness**	**0.117**	**0.053**	**0.180**	**1019**	**0.0013**
	Neophobia	0.030	-0.036	0.095	1017	0.3760
	Bodyweight	-0.195	-0.495	0.100	995	0.1978
	Fasting period	-0.186	-1.654	1.291	1036	0.8029
	Captive status: wild	-0.098	-0.392	0.198	993	0.4846
	Phylogeny	*0.042*	*0.000*	*0.096*	*827*	
	Species	*0.007*	*0.000*	*0.025*	*726*	
	Capture site	*0.008*	*0.000*	*0.025*	*971*	
	(Intercept)	0.823	0.530	1.127	1001	0.0010
	**Song repertoire**	**-0.174**	**-0.323**	**-0.030**	**1005**	**0.0217**
	**Shyness**	**0.107**	**0.044**	**0.170**	**1024**	**0.0016**
	Phylogeny	*0.039*	*0.000*	*0.096*	*704*	
	Species	*0.011*	*0.000*	*0.032*	*664*	
	Capture site	*0.007*	*0.000*	*0.021*	*989*	
Associative learning	(Intercept)	0.262	-1.306	1.810	1015	0.7428
	Song repertoire	0.049	-0.064	0.160	1006	0.3816
	Shyness	-0.013	-0.078	0.052	1012	0.6956
	**Neophobia**	**0.075**	**0.009**	**0.142**	**1023**	**0.0284**
	Bodyweight	-0.243	-0.504	0.014	991	0.0647
	Fasting period	1.001	-0.430	2.440	1020	0.1779
	Captive status: wild	0.078	-0.138	0.293	1008	0.4608
	Phylogeny	*0.018*	*0.000*	*0.046*	*921*	
	Species	*0.004*	*0.000*	*0.013*	*919*	
	Capture site	*0.003*	*0.000*	*0.008*	*965*	
	(Intercept)	1.183	0.975	1.400	1014	0.0010
	Song repertoire	0.024	-0.078	0.126	1008	0.6366
	**Neophobia**	**0.078**	**0.015**	**0.141**	**990**	**0.0153**
	Phylogeny	*0.016*	*0.000*	*0.039*	*929*	
	Species	*0.004*	*0.000*	*0.011*	*930*	
	Capture site	*0.002*	*0.000*	*0.007*	*990*	

Reversal learning	(Intercept)	1.477	0.016	2.947	1005	0.0510
	Song repertoire	0.070	-0.098	0.239	976	0.3945
	**Shyness**	**-0.094**	**-0.152**	**-0.036**	**997**	**0.0020**
	Neophobia	0.008	-0.052	0.068	990	0.7971
	**Bodyweight**	**-0.368**	**-0.683**	**-0.056**	**1020**	**0.0205**
	Fasting period	0.491	-0.821	1.789	1012	0.4657
	Captive status: wild	0.114	-0.183	0.413	942	0.4448
	Phylogeny	*0.051*	*0.000*	*0.129*	*679*	
	Species	*0.012*	*0.000*	*0.036*	*605*	
	Capture site	*0.003*	*0.000*	*0.009*	*994*	
	(Intercept)	2.064	1.575	2.569	1005	0.0010
	Song repertoire	0.066	-0.092	0.227	1001	0.4115
	**Shyness**	**-0.093**	**-0.150**	**-0.036**	**1002**	**0.0021**
	**Bodyweight**	**-0.288**	**-0.544**	**-0.036**	**1007**	**0.0238**
	Phylogeny	*0.056*	*0.000*	*0.126*	*748*	
	Species	*0.009*	*0.000*	*0.030*	*625*	
	Capture site	*0.003*	*0.000*	*0.009*	*998*	
Self-control	(Intercept)	1.660	0.203	3.102	1050	0.0273
	Song repertoire	0.010	-0.127	0.148	1014	0.8774
	**Shyness**	-0.114	-0.171	-0.056	1009	**0.0011**
	Neophobia	-0.008	-0.068	0.053	997	0.8037
	**Bodyweight**	0.416	0.146	0.686	1017	**0.0029**
	Fasting period	-0.851	-2.162	0.445	1039	0.2047
	Captive status: wild	0.131	-0.114	0.377	1027	0.2783
	Phylogeny	*0.027*	*0.000*	*0.079*	*727*	
	Species	*0.011*	*0.000*	*0.029*	*789*	
	Capture site	*0.002*	*0.000*	*0.006*	*956*	
	(Intercept)	0.858	0.421	1.296	979	0.0019
	Song repertoire	0.003	-0.144	0.148	1015	0.9554
	**Shyness**	**-0.109**	**-0.166**	**-0.051**	**1018**	**0.0012**
	**Bodyweight**	**0.348**	**0.115**	**0.589**	**996**	**0.0034**
	Phylogeny	*0.031*	*0.000*	*0.094*	*646*	
	Species	*0.013*	*0.000*	*0.035*	*731*	
	Capture site	*0.002*	*0.000*	*0.007*	*977*	
**Relative brain size**	(Intercept)	-0.824	-4.465	2.673	501	0.6920
	**Song repertoire**	**1.450**	**0.144**	**2.763**	**635**	**0.0331**
	Shyness	0.000	-0.027	0.027	493	0.9681
	Neophobia	0.000	-0.030	0.030	447	0.9637
	Captive status: wild	0.860	-1.087	2.815	943	0.3684
	Phylogeny	*5.551*	*0.300*	*11.590*	*180*	
	Species	*0.521*	*0.000*	*2.091*	*192*	
	(Intercept)	-0.050	-3.180	2.906	321	0.7454
	**Song repertoire**	**1.373**	**0.114**	**2.628**	**731**	**0.0362**
	Phylogeny	*5.803*	*0.358*	*11.485*	*230*	
	Species	*0.446*	*0.000*	*1.938*	*205*	

B) Call repertoire

Dependent variable	Independent variable	Post mean	$l_{95\%}$ CI	$u_{95\%}$ CI	Eff samp	pMCMC
**Problem-solving**	(Intercept)	1.035	-0.546	2.638	999	0.2047
	**Call repertoire**	-0.173	-0.327	-0.020	995	**0.0303**
	**Shyness**	0.124	0.060	0.188	1012	**0.0011**
	Neophobia	0.029	-0.037	0.095	1017	0.3916
	Bodyweight	-0.275	-0.556	0.008	999	0.0568
	Fasting period	0.169	-1.304	1.609	1000	0.8190
	Captive status: wild	0.005	-0.290	0.303	1047	0.9673
	Phylogeny	*0.033*	*0.000*	*0.086*	*721*	
	Species	*0.009*	*0.000*	*0.027*	*743*	
	Capture site	*0.007*	*0.000*	*0.024*	*952*	
	(Intercept)	0.855	0.565	1.153	1013	0.0010
	**Call repertoire**	**-0.187**	**-0.342**	**-0.032**	**987**	**0.0216**
	**Shyness**	**0.116**	**0.053**	**0.179**	**1015**	**0.0013**
	Phylogeny	*0.030*	*0.000*	*0.091*	*610*	
	Species	*0.014*	*0.000*	*0.034*	*717*	
	Capture site	*0.005*	*0.000*	*0.017*	*1000*	
Associative learning	(Intercept)	0.222	-1.340	1.767	1020	0.7785
	Call repertoire	-0.054	-0.168	0.060	1012	0.3322
	Shyness	-0.015	-0.079	0.050	1003	0.6544
	**Neophobia**	**0.076**	**0.010**	**0.143**	**1014**	**0.0267**
	Bodyweight	-0.227	-0.474	0.025	1019	0.0747
	Fasting period	1.088	-0.364	2.543	1017	0.1469
	Captive status: wild	0.093	-0.124	0.310	1006	0.3853
	Phylogeny	*0.018*	*0.000*	*0.044*	*956*	
	Species	*0.004*	*0.000*	*0.012*	*948*	
	Capture site	*0.003*	*0.000*	*0.008*	*970*	
	(Intercept)	1.244	1.023	1.473	1009	0.0010
	Call repertoire	-0.038	-0.141	0.068	1007	0.4620
	**Neophobia**	**0.079**	**0.017**	**0.142**	**1006**	**0.0141**
	Phylogeny	*0.016*	*0.000*	*0.039*	*937*	
	Species	*0.003*	*0.000*	*0.010*	*947*	
	Capture site	*0.002*	*0.000*	*0.007*	*970*	
Reversal learning	(Intercept)	1.499	0.025	2.969	1014	0.0477
	Call repertoire	-0.049	-0.221	0.125	1006	0.5563
	**Shyness**	**-0.095**	**-0.153**	**-0.037**	**1009**	**0.0021**
	Neophobia	0.009	-0.051	0.070	1001	0.7702
	**Bodyweight**	**-0.334**	**-0.637**	**-0.032**	**1015**	**0.0288**
	Fasting period	0.504	-0.815	1.821	1028	0.4564
	Captive status: wild	0.128	-0.181	0.445	881	0.4183
	Phylogeny	*0.045*	*0.000*	*0.122*	*641*	
	Species	*0.014*	*0.000*	*0.038*	*633*	

	Capture site	*0.003*	*0.000*	*0.009*	*975*	
	(Intercept)	2.098	1.599	2.609	995	0.0010
	Call repertoire	-0.029	-0.194	0.140	1015	0.7206
	**Shyness**	**-0.094**	**-0.152**	**-0.037**	**1012**	**0.0019**
	**Bodyweight**	**-0.257**	**-0.507**	**-0.016**	**1013**	**0.0375**
	Phylogeny	*0.053*	*0.000*	*0.123*	*739*	
	Species	*0.010*	*0.000*	*0.032*	*636*	
	Capture site	*0.003*	*0.000*	*0.009*	*975*	
Self-control	(Intercept)	1.642	0.188	3.104	1012	0.0297
	Call repertoire	-0.017	-0.163	0.129	1011	0.8031
	**Shyness**	-0.114	-0.173	-0.057	1014	**0.0011**
	Neophobia	-0.008	-0.068	0.053	1010	0.8084
	**Bodyweight**	0.418	0.157	0.684	1005	**0.0024**
	Fasting period	-0.824	-2.143	0.490	1005	0.2265
	Captive status: wild	0.137	-0.113	0.389	1007	0.2720
	Phylogeny	*0.026*	*0.000*	*0.078*	*693*	
	Species	*0.011*	*0.000*	*0.029*	*786*	
	Capture site	*0.002*	*0.000*	*0.006*	*956*	
	(Intercept)	0.865	0.416	1.313	1007	0.0024
	Call repertoire	-0.009	-0.162	0.147	987	0.8932
	**Shyness**	**-0.109**	**-0.166**	**-0.053**	**1019**	**0.0011**
	**Bodyweight**	**0.350**	**0.120**	**0.587**	**1005**	**0.0025**
	Phylogeny	*0.032*	*0.000*	*0.096*	*663*	
	Species	*0.013*	*0.000*	*0.034*	*744*	
	Capture site	*0.002*	*0.000*	*0.007*	*982*	
**Relative brain size**	(Intercept)	-0.650	-4.014	2.531	505	0.7393
	**Call repertoire**	1.742	0.335	3.160	635	**0.0182**
	Shyness	0.000	-0.028	0.027	497	0.9666
	Neophobia	0.000	-0.030	0.030	467	0.9643
	Captive status: wild	0.016	-2.109	2.161	581	0.8469
	Phylogeny	*4.357*	*0.322*	*10.652*	*145*	
	Species	*0.803*	*0.000*	*2.251*	*187*	
	(Intercept)	-0.385	-3.344	2.091	221	0.7443
	**Call repertoire**	**1.702**	**0.392**	**2.989**	**594**	**0.0132**
	Phylogeny	*3.571*	*0.131*	*9.167*	*143*	
	Species	*0.814*	*0.000*	*2.203*	*149*	

Table S7. MCMCglmm phylogenetic models assessing relationships between all measured behaviors, brain size, and vocal learning complexity (PC1). Vocal learning complexity, personality traits and experimental conditions (except for brain size) were included as fixed effects, and species, phylogeny and capture site as random effects. All variables were included in initial models (above dashed line), and then they were run with only vocal learning complexity and the significant covariate(s) when applicable (below dashed line). All measured behaviors (but not brain size) are expressed in logged trials; therefore, higher numbers represent lower performance. Significant effects are highlighted in bold and random effects are in italics (n = 23 species, 214 individuals).

Dependent variable	Independent variable	Post mean	$l_{95\%}$ CI	$u_{95\%}$ CI	Eff samp	pMCMC
**Problem-solving**	(Intercept)	0.906	-0.672	2.493	1005	0.2688
	**Vocal learning complexity**	-0.079	-0.129	-0.031	1007	**0.0039**
	**Shyness**	0.127	0.064	0.191	1023	**0.0011**
	Neophobia	0.024	-0.041	0.088	992	0.4741
	Bodyweight	-0.144	-0.425	0.136	987	0.3136
	Fasting period	0.028	-1.413	1.462	1021	0.9629
	Captive status: wild	-0.075	-0.362	0.214	1034	0.5775
	Phylogeny	*0.025*	*0.000*	*0.067*	*767*	
	Species	*0.007*	*0.000*	*0.021*	*789*	
	Capture site	*0.009*	*0.000*	*0.030*	*978*	
	(Intercept)	0.668	0.456	0.889	1026	0.0010
	**Vocal learning complexity**	**-0.090**	**-0.134**	**-0.046**	**1019**	**0.0012**
	**Shyness**	**0.121**	**0.058**	**0.183**	**1024**	**0.0011**
	Phylogeny	*0.019*	*0.000*	*0.056*	*726*	
	Species	*0.009*	*0.000*	*0.021*	*833*	
	Capture site	*0.007*	*0.000*	*0.023*	*970*	
Associative learning	(Intercept)	0.290	-1.275	1.860	1026	0.7154
	Vocal learning complexity	-0.012	-0.055	0.033	1008	0.5876
	Shyness	-0.015	-0.079	0.050	1001	0.6580
	**Neophobia**	**0.075**	**0.008**	**0.141**	**1035**	**0.0292**
	Bodyweight	-0.196	-0.457	0.066	1028	0.1382
	Fasting period	0.971	-0.477	2.416	1025	0.1918
	Captive status: wild	0.066	-0.146	0.282	1021	0.5314
	Phylogeny	*0.019*	*0.000*	*0.047*	*955*	
	Species	*0.004*	*0.000*	*0.013*	*925*	
	Capture site	*0.003*	*0.000*	*0.008*	*970*	
	(Intercept)	1.209	1.013	1.411	1002	0.0010
	Vocal learning complexity	-0.016	-0.054	0.021	1015	0.3768
	**Neophobia**	**0.079**	**0.017**	**0.142**	**1005**	**0.0141**
	Phylogeny	*0.017*	*0.001*	*0.039*	*953*	
	Species	*0.003*	*0.000*	*0.010*	*944*	
	Capture site	*0.002*	*0.000*	*0.006*	*970*	

Reversal learning	(Intercept)	1.460	-0.025	2.940	996	0.0565
	Vocal learning complexity	-0.017	-0.080	0.048	997	0.5714
	**Shyness**	**-0.095**	**-0.154**	**-0.037**	**1017**	**0.0022**
	Neophobia	0.008	-0.053	0.069	1008	0.7955
	Bodyweight	-0.306	-0.624	0.008	1012	0.0550
	Fasting period	0.480	-0.839	1.778	1007	0.4787
	Captive status: wild	0.109	-0.191	0.417	913	0.4768
	Phylogeny	*0.044*	*0.000*	*0.119*	*635*	
	Species	*0.013*	*0.000*	*0.037*	*662*	
	Capture site	*0.003*	*0.000*	*0.009*	*985*	
	(Intercept)	1.706	1.391	2.048	996	0.0010
	Vocal learning complexity	-0.037	-0.093	0.021	1026	0.1978
	**Shyness**	**-0.094**	**-0.152**	**-0.036**	**1023**	**0.0023**
	Phylogeny	*0.043*	*0.000*	*0.113*	*670*	
	Species	*0.012*	*0.000*	*0.034*	*650*	
	Capture site	*0.003*	*0.000*	*0.009*	*967*	
Self-control	(Intercept)	1.572	0.086	3.024	1020	0.0385
	Vocal learning complexity	-0.018	-0.072	0.035	980	0.4928
	**Shyness**	-0.114	-0.172	-0.057	992	**0.0010**
	Neophobia	-0.008	-0.068	0.052	1010	0.7975
	**Bodyweight**	0.445	0.175	0.724	1024	**0.0019**
	Fasting period	-0.810	-2.121	0.494	1019	0.2314
	Captive status: wild	0.131	-0.114	0.377	1002	0.2816
	Phylogeny	*0.024*	*0.000*	*0.075*	*683*	
	Species	*0.012*	*0.000*	*0.030*	*790*	
	Capture site	*0.002*	*0.000*	*0.006*	*982*	
	(Intercept)	0.799	0.353	1.259	971	0.0033
	Vocal learning complexity	-0.023	-0.080	0.034	1003	0.4057
	**Shyness**	**-0.109**	**-0.166**	**-0.052**	**1020**	**0.0012**
	**Bodyweight**	**0.386**	**0.143**	**0.638**	**986**	**0.0022**
	Phylogeny	*0.028*	*0.000*	*0.090*	*638*	
	Species	*0.014*	*0.000*	*0.036*	*752*	
	Capture site	*0.002*	*0.000*	*0.007*	*966*	
**Relative brain size**	(Intercept)	0.575	-2.515	3.514	470	0.6075
	**Vocal learning complexity**	**0.681**	**0.233**	**1.142**	**589**	**0.0039**
	Shyness	0.000	-0.027	0.027	506	0.9582
	Neophobia	0.000	-0.030	0.030	439	0.9609
	Captive status: wild	0.584	-1.245	2.448	785	0.5269
	Phylogeny	*4.117*	*0.233*	*9.423*	*166*	
	Species	*0.600*	*0.000*	*1.897*	*165*	
	(Intercept)	1.145	-1.521	3.496	297	0.3876
	**Vocal learning complexity**	**0.678**	**0.242**	**1.119**	**573**	**0.0037**
	Phylogeny	*4.052*	*0.245*	*8.907*	*184*	
	Species	*0.495*	*0.000*	*1.685*	*198*	

Table S8. MCMCglmm phylogenetic models assessing relationships between all measured behaviors, brain size, and vocal learning complexity (PC1) in the 15 species with larger sample sizes. Vocal learning complexity, personality traits and experimental conditions (except for brain size) were included as fixed effects, and species, phylogeny and capture site as random effects. All variables were included in initial models (above dashed line), and then they were run with only vocal learning complexity and the significant covariate(s) when applicable (below dashed line). All measured behaviors (but not brain size) are expressed in logged trials; therefore, higher numbers represent lower performance. Significant effects are highlighted in bold and random effects are in italics (n = 15 species, 203 individuals).

Dependent variable	Independent variable	Post mean	$l_{95\%}$ CI	$u_{95\%}$ CI	Eff samp	pMCMC
**Problem-solving**	(Intercept)	0.745	-0.888	2.374	1013	0.3769
	Vocal learning complexity	-0.062	-0.126	0.002	996	0.0644
	**Shyness**	**0.123**	**0.056**	**0.189**	**1015**	**0.0012**
	Neophobia	0.024	-0.044	0.093	994	0.4867
	Bodyweight	-0.184	-0.534	0.153	985	0.2839
	Fasting period	0.186	-1.289	1.686	1015	0.8085
	Captive status: wild	-0.060	-0.374	0.258	1006	0.6741
	Phylogeny	*0.024*	*0.000*	*0.066*	*914*	
	Species	*0.009*	*0.000*	*0.029*	*920*	
	Capture site	*0.010*	*0.000*	*0.033*	*980*	
	(Intercept)	0.676	0.481	0.883	997	0.0010
	**Vocal learning complexity**	**-0.078**	**-0.133**	**-0.023**	**1015**	**0.0122**
	**Shyness**	**0.118**	**0.052**	**0.183**	**1013**	**0.0014**
	Phylogeny	*0.019*	*0.000*	*0.055*	*892*	
	Species	*0.010*	*0.000*	*0.027*	*947*	
	Capture site	*0.008*	*0.000*	*0.026*	*956*	
Associative learning	(Intercept)	0.143	-1.455	1.755	1004	0.8617
	Vocal learning complexity	-0.005	-0.056	0.048	1001	0.8372
	Shyness	-0.010	-0.078	0.057	1023	0.7760
	**Neophobia**	**0.081**	**0.011**	**0.150**	**1015**	**0.0243**
	Bodyweight	-0.274	-0.582	0.030	1001	0.0700
	Fasting period	1.164	-0.340	2.644	990	0.1306
	Captive status: wild	0.079	-0.154	0.311	1000	0.4915
	Phylogeny	*0.017*	*0.000*	*0.044*	*979*	
	Species	*0.005*	*0.000*	*0.018*	*957*	
	Capture site	*0.002*	*0.000*	*0.007*	*973*	
	(Intercept)	1.205	1.054	1.360	1021	0.0010
	Vocal learning complexity	-0.015	-0.058	0.029	1036	0.4833
	**Neophobia**	**0.083**	**0.018**	**0.148**	**1018**	**0.0147**
	Phylogeny	*0.014*	*0.000*	*0.034*	*978*	
	Species	*0.004*	*0.000*	*0.012*	*969*	
	Capture site	*0.002*	*0.000*	*0.006*	*982*	
**Reversal learning**	(Intercept)	1.519	0.016	3.003	1000	0.0486

	Vocal learning complexity	-0.022	-0.098	0.053	1036	0.5193
	**Shyness**	**-0.091**	**-0.150**	**-0.031**	**1005**	**0.0039**
	Neophobia	0.013	-0.048	0.075	1014	0.6831
	Bodyweight	-0.269	-0.636	0.086	1016	0.1328
	Fasting period	0.433	-0.903	1.779	997	0.5288
	Captive status: wild	0.041	-0.268	0.356	985	0.8049
	Phylogeny	*0.042*	*0.000*	*0.103*	*946*	
	Species	*0.011*	*0.000*	*0.038*	*866*	
	Capture site	*0.004*	*0.000*	*0.011*	*984*	
	(Intercept)	1.710	1.468	1.958	1023	0.0010
	Vocal learning complexity	-0.040	-0.105	0.025	1013	0.2119
	**Shyness**	**-0.092**	**-0.151**	**-0.034**	**1021**	**0.0026**
	Phylogeny	*0.037*	*0.001*	*0.085*	*968*	
	Species	*0.009*	*0.000*	*0.029*	*899*	
	Capture site	*0.003*	*0.000*	*0.010*	*996*	
Self-control	(Intercept)	1.401	-0.091	2.899	1007	0.0699
	Vocal learning complexity	-0.010	-0.076	0.055	1011	0.7699
	**Shyness**	**-0.109**	**-0.169**	**-0.050**	**1029**	**0.0012**
	Neophobia	-0.006	-0.067	0.057	1006	0.8534
	**Bodyweight**	**0.449**	**0.114**	**0.807**	**1030**	**0.0076**
	Fasting period	-0.668	-1.995	0.670	1011	0.3248
	Captive status: wild	0.097	-0.169	0.363	1008	0.4455
	Phylogeny	*0.025*	*0.000*	*0.071*	*913*	
	Species	*0.013*	*0.000*	*0.037*	*942*	
	Capture site	*0.002*	*0.000*	*0.006*	*978*	
	(Intercept)	0.766	0.269	1.235	1005	0.0098
	Vocal learning complexity	-0.010	-0.079	0.056	1026	0.7641
	**Shyness**	**-0.104**	**-0.162**	**-0.045**	**1002**	**0.0013**
	**Bodyweight**	**0.387**	**0.094**	**0.706**	**1007**	**0.0069**
	Phylogeny	*0.027*	*0.000*	*0.077*	*901*	
	Species	*0.014*	*0.000*	*0.039*	*949*	
	Capture site	*0.002*	*0.000*	*0.006*	*987*	
**Relative brain size**	(Intercept)	-0.164	-2.397	2.162	516	0.7392
	**Vocal learning complexity**	**0.566**	**0.090**	**1.058**	**720**	**0.0220**
	Shyness	0.000	-0.034	0.034	435	0.9609
	Neophobia	0.001	-0.039	0.040	355	0.9546
	Captive status: wild	1.561	-0.529	3.638	459	0.1529
	Phylogeny	*1.125*	*0.000*	*4.982*	*269*	
	Species	*1.213*	*0.000*	*2.833*	*346*	
	(Intercept)	1.179	-0.764	3.061	881	0.1857
	**Vocal learning complexity**	**0.623**	**0.093**	**1.162**	**852**	**0.0256**
	Phylogeny	*2.449*	*0.000*	*6.735*	*237*	
	Species	*0.838*	*0.000*	*2.768*	*224*	

Movie captions

Movie S1. Lid-pulling problem-solving task.

Movie S2. Lid-flipping problem-solving task.

Movie S3. Lid-piercing problem-solving task.

Movie S4. Stick-pulling problem-solving task.

Movie S5. Detour reaching task (failed trial) that assessed self-control.

Movie S6. Detour reaching task (succeeded trial) that assessed self-control.

Movie S7. Discrimination learning task that assessed associative and reversal learning.

References and Notes

1. E. D. Jarvis, Evolution of vocal learning and spoken language. *Science* **366**, 50–54 (2019). doi:10.1126/science.aax0287 Medline

2. V. M. Janik, P. J. B. Slater, The different roles of social learning in vocal communication. *Anim. Behav.* **60**, 1–11 (2000). doi:10.1006/anbe.2000.1410 Medline

3. J. O. Van Horik, N. S. Clayton, N. J. Emery, "Convergent evolution of cognition in corvids, apes and other animals" in *The Oxford Handbook of Comparative Evolutionary Psychology*, T. K. Shackelford, J. Vonk, Eds. (Oxford Univ. Press, 2012).

4. S. M. Reader, K. N. Laland, Eds., *Animal Innovation* (Oxford Univ. Press, ed. 1, 2003).

5. W. A. Searcy, S. Nowicki, Birdsong learning, avian cognition and the evolution of language. *Anim. Behav.* **151**, 217–227 (2019). doi:10.1016/j.anbehav.2019.01.015

6. N. J. Boogert, L.-A. Giraldeau, L. Lefebvre, Song complexity correlates with learning ability in zebra finch males. *Anim. Behav.* **76**, 1735–1741 (2008). doi:10.1016/j.anbehav.2008.08.009

7. N. J. Boogert, R. C. Anderson, S. Peters, W. A. Searcy, S. Nowicki, Song repertoire size in male song sparrows correlates with detour reaching, but not with other cognitive measures. *Anim. Behav.* **81**, 1209–1216 (2011). doi:10.1016/j.anbehav.2011.03.004

8. C. N. Templeton, K. N. Laland, N. J. Boogert, Does song complexity correlate with problem-solving performance in flocks of zebra finches? *Anim. Behav.* **92**, 63–71 (2014). doi:10.1016/j.anbehav.2014.03.019

9. R. C. Anderson, W. A. Searcy, S. Peters, M. Hughes, A. L. DuBois, S. Nowicki, Song learning and cognitive ability are not consistently related in a songbird. *Anim. Cogn.* **20**, 309–320 (2017). doi:10.1007/s10071-016-1053-7 Medline

10. A. L. DuBois, S. Nowicki, S. Peters, K. D. Rivera-Cáceres, W. A. Searcy, Song is not a reliable signal of general cognitive ability in a songbird. *Anim. Behav.* **137**, 205–213 (2018). doi:10.1016/j.anbehav.2018.01.020

11. K. B. Sewall, J. A. Soha, S. Peters, S. Nowicki, Potential trade-off between vocal ornamentation and spatial ability in a songbird. *Biol. Lett.* **9**, 20130344 (2013). doi:10.1098/rsbl.2013.0344 Medline

12. T. M. Farrell, K. Weaver, Y.-S. An, S. A. MacDougall-Shackleton, Song bout length is indicative of spatial learning in European starlings. *Behav. Ecol.* **23**, 101–111 (2012). doi:10.1093/beheco/arr162

13. K. L. Schmidt, S. D. Moore, E. A. MacDougall-Shackleton, S. A. MacDougall-Shackleton, Early-life stress affects song complexity, song learning and volume of the brain nucleus RA in adult male song sparrows. *Anim. Behav.* **86**, 25–35 (2013). doi:10.1016/j.anbehav.2013.03.036

14. S. C. Vernes, B. P. Kriengwatana, V. C. Beeck, J. Fischer, P. L. Tyack, C. Ten Cate, V. M. Janik, The multi-dimensional nature of vocal learning. *Philos. Trans. R. Soc. Lond. Ser. B* **376**, 20200236 (2021). doi:10.1098/rstb.2020.0236 Medline

15. M. Wirthlin, E. F. Chang, M. Knörnschild, L. A. Krubitzer, C. V. Mello, C. T. Miller, A. R. Pfenning, S. C. Vernes, O. Tchernichovski, M. M. Yartsev, A modular approach to vocal

learning: Disentangling the diversity of a complex behavioral trait. *Neuron* **104**, 87–99 (2019). doi:10.1016/j.neuron.2019.09.036 Medline

16. W. A. Searcy, J. Soha, S. Peters, S. Nowicki, Variation in vocal production learning across songbirds. *Philos. Trans. R. Soc. Lond. Ser. B* **376**, 20200257 (2021). doi:10.1098/rstb.2020.0257 Medline

17. P. Rundstrom, N. Creanza, Song learning and plasticity in songbirds. *Curr. Opin. Neurobiol.* **67**, 228–239 (2021). doi:10.1016/j.conb.2021.02.003 Medline

18. C. M. Robinson, K. T. Snyder, N. Creanza, Correlated evolution between repertoire size and song plasticity predicts that sexual selection on song promotes open-ended learning. *eLife* **8**, e44454 (2019). doi:10.7554/eLife.44454 Medline

19. J.-N. Audet, Neurobiological and ecological correlates of avian innovation. *Integr. Comp. Biol.* **60**, 955–966 (2020). doi:10.1093/icb/icaa107 Medline

20. J.-N. Audet, L. Lefebvre, What's flexible in behavioral flexibility? *Behav. Ecol.* **28**, 943–947 (2017). doi:10.1093/beheco/arx007

21. F. Sayol, D. Sol, A. L. Pigot, Brain size and life history interact to predict urban tolerance in birds. *Front. Ecol. Evol.* **8**, 58 (2020). doi:10.3389/fevo.2020.00058

22. L. Lefebvre, S. M. Reader, D. Sol, Brains, innovations and evolution in birds and primates. *Brain Behav. Evol.* **63**, 233–246 (2004). doi:10.1159/000076784 Medline

23. S. Benson-Amram, B. Dantzer, G. Stricker, E. M. Swanson, K. E. Holekamp, Brain size predicts problem-solving ability in mammalian carnivores. *Proc. Natl. Acad. Sci. U.S.A.* **113**, 2532–2537 (2016). doi:10.1073/pnas.1505913113 Medline

24. D. Sol, S. Olkowicz, F. Sayol, M. Kocourek, Y. Zhang, L. Marhounová, C. Osadnik, E. Corssmit, J. Garcia-Porta, T. E. Martin, L. Lefebvre, P. Němec, Neuron numbers link innovativeness with both absolute and relative brain size in birds. *Nat. Ecol. Evol.* **6**, 1381–1389 (2022). doi:10.1038/s41559-022-01815-x Medline

25. T. J. Devoogd, J. R. Krebs, S. D. Healy, A. Purvis, Relations between song repertoire size and the volume of brain nuclei related to song: Comparative evolutionary analyses amongst oscine birds. *Proc. Biol. Sci.* **254**, 75–82 (1993). doi:10.1098/rspb.1993.0129 Medline

26. L. Z. Garamszegi, M. Eens, J. Erritzøe, A. P. Møller, Sexually size dimorphic brains and song complexity in passerine birds. *Behav. Ecol.* **16**, 335–345 (2005). doi:10.1093/beheco/arh167

27. S. A. Macdougall-Shackleton, "Sexual selection and the evolution of song repertoires" in *Current Ornithology*, V. Nolan, E. D. Ketterson, C. F. Thompson, Eds. (Springer, 1997), pp. 81–124.

28. J.-N. Audet, S. Ducatez, L. Lefebvre, The town bird and the country bird: Problem solving and immunocompetence vary with urbanization. *Behav. Ecol.* **27**, 637–644 (2016). doi:10.1093/beheco/arv201

29. J. L. Dowling, D. A. Luther, P. P. Marra, Comparative effects of urban development and anthropogenic noise on bird songs. *Behav. Ecol.* **23**, 201–209 (2012). doi:10.1093/beheco/arr176

30. P. Marler, Bird calls: Their potential for behavioral neurobiology. *Ann. N. Y. Acad. Sci.* **1016**, 31–44 (2004). doi:10.1196/annals.1298.034 Medline

31. K. B. Sewall, A. M. Young, T. F. Wright, Social calls provide novel insights into the evolution of vocal learning. *Anim. Behav.* **120**, 163–172 (2016). doi:10.1016/j.anbehav.2016.07.031 Medline

32. S. Olkowicz, M. Kocourek, R. K. Lučan, M. Porteš, W. T. Fitch, S. Herculano-Houzel, P. Němec, Birds have primate-like numbers of neurons in the forebrain. *Proc. Natl. Acad. Sci. U.S.A.* **113**, 7255–7260 (2016). doi:10.1073/pnas.1517131113 Medline

33. G. Feenders, M. Liedvogel, M. Rivas, M. Zapka, H. Horita, E. Hara, K. Wada, H. Mouritsen, E. D. Jarvis, Molecular mapping of movement-associated areas in the avian brain: A motor theory for vocal learning origin. *PLOS ONE* **3**, e1768 (2008). doi:10.1371/journal.pone.0001768 Medline

34. A. D. Patel, Vocal learning as a preadaptation for the evolution of human beat perception and synchronization. *Philos. Trans. R. Soc. Lond. Ser. B* **376**, 20200326 (2021). doi:10.1098/rstb.2020.0326 Medline

35. M. Chakraborty, E. D. Jarvis, Brain evolution by brain pathway duplication. *Philos. Trans. R. Soc. Lond. Ser. B* **370**, 20150056 (2015). doi:10.1098/rstb.2015.0056 Medline

36. O. Güntürkün, T. Bugnyar, Cognition without cortex. *Trends Cogn. Sci.* **20**, 291–303 (2016). doi:10.1016/j.tics.2016.02.001 Medline

37. F. S. Livingston, R. Mooney, Development of intrinsic and synaptic properties in a forebrain nucleus essential to avian song learning. *J. Neurosci.* **17**, 8997–9009 (1997). doi:10.1523/JNEUROSCI.17-23-08997.1997 Medline

38. M. E. Basham, E. J. Nordeen, K. W. Nordeen, Blockade of NMDA receptors in the anterior forebrain impairs sensory acquisition in the zebra finch (*Poephila guttata*). *Neurobiol. Learn. Mem.* **66**, 295–304 (1996). doi:10.1006/nlme.1996.0071 Medline

39. J.-N. Audet, L. Kayello, S. Ducatez, S. Perillo, L. Cauchard, J. T. Howard, L. A. O'Connell, E. D. Jarvis, L. Lefebvre, Divergence in problem-solving skills is associated with differential expression of glutamate receptors in wild finches. *Sci. Adv.* **4**, eaao6369 (2018). doi:10.1126/sciadv.aao6369 Medline

40. T. W. Deacon, *Symbolic Species* (Norton, 1998).

41. Y. Toda, M.-C. Ko, Q. Liang, E. T. Miller, A. Rico-Guevara, T. Nakagita, A. Sakakibara, K. Uemura, T. Sackton, T. Hayakawa, S. Y. W. Sin, Y. Ishimaru, T. Misaka, P. Oteiza, J. Crall, S. V. Edwards, W. Buttemer, S. Matsumura, M. W. Baldwin, Early origin of sweet perception in the songbird radiation. *Science* **373**, 226–231 (2021). doi:10.1126/science.abf6505 Medline

42. C. R. Cooney, J. A. Bright, E. J. R. Capp, A. M. Chira, E. C. Hughes, C. J. A. Moody, L. O. Nouri, Z. K. Varley, G. H. Thomas, Mega-evolutionary dynamics of the adaptive radiation of birds. *Nature* **542**, 344–347 (2017). doi:10.1038/nature21074 Medline

43. F. Sayol, P. A. Downing, A. N. Iwaniuk, J. Maspons, D. Sol, Predictable evolution towards larger brains in birds colonizing oceanic islands. *Nat. Commun.* **9**, 2820 (2018). doi:10.1038/s41467-018-05280-8 Medline

44. J.-N. Audet, M. Couture, E. D. Jarvis, Songbird species that display more-complex vocal learning are better problem-solvers and have larger brains. Dryad (2023); https://doi.org/10.5061/dryad.tb2rbp06n.

45. J.-N. Audet, S. Ducatez, L. Lefebvre, Morphological and molecular sexing of the monochromatic Barbados bullfinch, *Loxigilla barbadensis*. *Zool. Sci.* **31**, 687–691 (2014). doi:10.2108/zs140025 Medline

46. R. Griffiths, M. C. Double, K. Orr, R. J. Dawson, A DNA test to sex most birds. *Mol. Ecol.* **7**, 1071–1075 (1998). doi:10.1046/j.1365-294x.1998.00389.x Medline

47. C. W. Dobson, R. E. Lemon, Re-examination of monotony threshold hypothesis in bird song. *Nature* **257**, 126–128 (1975). doi:10.1038/257126a0

48. A. F. Read, D. M. Weary, The evolution of bird song: Comparative analyses. *Philos. Trans. R. Soc. Lond. Ser. B* **338**, 165–187 (1992). doi:10.1098/rstb.1992.0137

49. D. J. Mountjoy, R. E. Lemon, Extended song learning in wild European starlings. *Anim. Behav.* **49**, 357–366 (1995). doi:10.1006/anbe.1995.0048

50. P. G. Mota, G. C. Cardoso, Song organisation and patterns of variation in the serin (*Serinus serinus*). *Acta Ethol.* **3**, 141–150 (2001). doi:10.1007/s102110000034

51. V. M. Dziadosz, thesis, The Ohio State University (1977).

52. P. C. Mundinger, Call learning in the Carduelinae: Ethological and systematic considerations. *Syst. Zool.* **28**, 270–283 (1979). doi:10.2307/2412582

53. S. Ducatez, D. Sol, F. Sayol, L. Lefebvre, Behavioural plasticity is associated with reduced extinction risk in birds. *Nat. Ecol. Evol.* **4**, 788–793 (2020). doi:10.1038/s41559-020-1168-8 Medline

54. S. Lê, J. Josse, F. Husson, FactoMineR: An R package for multivariate analysis. *J. Stat. Softw.* **25**, 1–18 (2008). doi:10.18637/jss.v025.i01

55. C. D. Stahel, D. Megirian, S. C. Nicol, Sleep and metabolic rate in the little penguin, *Eudyptula minor*. *J. Comp. Physiol. B* **154**, 487–494 (1984). doi:10.1007/BF02515153

56. A. Ferretti, N. C. Rattenborg, T. Ruf, S. R. McWilliams, M. Cardinale, L. Fusani, Sleeping unsafely tucked in to conserve energy in a nocturnal migratory songbird. *Curr. Biol.* **29**, 2766–2772.e4 (2019). doi:10.1016/j.cub.2019.07.028 Medline

57. L. Amo, S. P. Caro, M. E. Visser, Sleeping birds do not respond to predator odour. *PLOS ONE* **6**, e27576 (2011). doi:10.1371/journal.pone.0027576 Medline

58. A. M. Fontvieille, R. Rising, M. Spraul, D. E. Larson, E. Ravussin, Relationship between sleep stages and metabolic rate in humans. *Am. J. Physiol.* **267**, E732–E737 (1994). doi:10.1152/ajpendo.1994.267.5.E732 Medline

59. P. E. Bickler, CO_2 balance of a heterothermic rodent: Comparison of sleep, torpor, and awake states. *Am. J. Physiol.* **246**, R49–R55 (1984). Medline

60. S. Ducatez, J. N. Audet, L. Lefebvre, Problem-solving and learning in Carib grackles: Individuals show a consistent speed-accuracy trade-off. *Anim. Cogn.* **18**, 485–496 (2015). doi:10.1007/s10071-014-0817-1 Medline

61. R Core Team, R: A language and environment for statistical computing (2021); https://www.R-project.org/.

62. M. W. Pennell, J. M. Eastman, G. J. Slater, J. W. Brown, J. C. Uyeda, R. G. FitzJohn, M. E. Alfaro, L. J. Harmon, geiger v2.0: An expanded suite of methods for fitting macroevolutionary models to phylogenetic trees. *Bioinformatics* **30**, 2216–2218 (2014). doi:10.1093/bioinformatics/btu181 Medline

63. J. D. Hadfield, MCMC methods for multi-response generalized linear mixed models: The MCMCglmm R package. *J. Stat. Softw.* **33**, 1–22 (2010). doi:10.18637/jss.v033.i02

64. P. C. Mundinger, Vocal imitation and individual recognition of finch calls. *Science* **168**, 480–482 (1970). doi:10.1126/science.168.3930.480 Medline

65. E. L. Coutlee, Vocalizations in the genus *Spinus*. *Anim. Behav.* **19**, 556–565 (1971). doi:10.1016/S0003-3472(71)80112-4

66. R. E. Lemon, R. Cotter, R. C. MacNally, S. Monette, Song repertoires and song sharing by american redstarts. *Condor* **87**, 457–470 (1985). doi:10.2307/1367942

67. R. E. Lemon, S. Monette, D. Roff, Song repertoires of american warblers (Parulinae): Honest advertising or assessment? *Ethology* **74**, 265–284 (1987). doi:10.1111/j.1439-0310.1987.tb00938.x

68. M. S. Ficken, Agonistic behavior and territory in the American redstart. *Auk* **79**, 607–632 (1962). doi:10.2307/4082642

69. E. T. Griffiths, S. C. Keen, M. Lanzone, A. Farnsworth, Can nocturnal flight calls of the migrating songbird, American redstart, encode sexual dimorphism and individual identity? *PLOS ONE* **11**, e0156578 (2016). doi:10.1371/journal.pone.0156578 Medline

70. S. L. Johnson, Do American robins acquire songs by both imitating and inventing? *Wilson J. Ornithol.* **118**, 341–352 (2006). doi:10.1676/05-048.1

71. A. C. Bent, *Life Histories of North American Thrushes, Kinglets, and Their Allies: Order Passeriformes* (United States National Museum Bulletin 196, US Government Printing Office, 1949).

72. D. Borror, Songs of the thrushes (Turdidae), wrens (Troglodytidae), and mockingbirds (Mimidae) of eastern North America. *Ohio J. Sci.* **64**, 195 (1964).

73. J. R. Benson, thesis, Oregon State University (1974).

74. L. L. Bloomfield, C. B. Sturdy, L. S. Phillmore, R. G. Weisman, Open-ended categorization of chick-a-dee calls by black-capped chickadees (*Poecile atricapilla*). *J. Comp. Psychol.* **117**, 290–301 (2003). doi:10.1037/0735-7036.117.3.290 Medline

75. D. E. Kroodsma, B. E. Byers, S. L. Halkin, C. Hill, D. Minis, J. R. Bolsinger, J.-A. Dawson, E. Donelan, J. Farrington, F. B. Gill, P. Houlihan, D. Innes, G. Keller, L. MacAulay, C. A. Marantz, J. Ortiz, P. K. Stoddard, K. Wilda, Geographic variation in black-capped chickadee songs and singing behavior. *Auk* **116**, 387–402 (1999). doi:10.2307/4089373

76. D. E. Gammon, M. C. Baker, Song repertoire evolution and acoustic divergence in a population of black-capped chickadees, *Poecile atricapillus*. *Anim. Behav.* **68**, 903–913 (2004). doi:10.1016/j.anbehav.2003.10.030

77. M. S. Ficken, R. W. Ficken, S. R. Witkin, Vocal repertoire of the black-capped chickadee. *Auk* **95**, 34–48 (1978). doi:10.2307/4085493

78. J. P. Hailman, M. S. Ficken, R. W. Ficken, The 'chick-a-dee' calls of *Parus atricapillus*: A recombinant system of animal communication compared with written English. *Semiotica* **56**, 191–224 (1985). doi:10.1515/semi.1985.56.3-4.191

79. A. C. Bent, *Life Histories of North American Jays, Crows and Titmice, Part I* (United States National Museum Bulletin 191, US Government Printing Office, 1946).

80. D. Brewer, thesis, Eastern Kentucky University (2016).

81. S. Conant, thesis, The University of Oklahoma (1972).

82. S. M. Cohen, thesis, University of Michigan (1977).

83. M. J. West, A. P. King, Song repertoire development in male cowbirds (*Molothrus ater*): Its relation to female assessment of song potency. *J. Comp. Psychol.* **100**, 296–303 (1986). doi:10.1037/0735-7036.100.3.296 Medline

84. L. Merrill, A. L. O'Loghlen, J. C. Wingfield, S. I. Rothstein, Linking a static signal to current condition: Song-repertoire size, corticosterone, and immunity in the brown-headed cowbird. *Condor* **115**, 434–441 (2013). doi:10.1525/cond.2013.120110

85. A. L. O'Loghlen, S. I. Rothstein, Ecological effects on song learning: Delayed development is widespread in wild populations of brown-headed cowbirds. *Anim. Behav.* **63**, 475–486 (2002). doi:10.1006/anbe.2001.1951

86. A. M. Dufty, R. McChrystal, Vocalizations and copulatory attempts in free-living brown-headed cowbirds (Vocalización e Intentos de Copulación en Individuos Silvestres de Molothrus ater). *J. Field Ornithol.* **63**, 16–25 (1992).

87. S. I. Rothstein, C. Farmer, J. Verner, "The structure and function of cowbird vocalizations and the use of playbacks to enhance cowbird detectability: Relations to potential censusing biases" in *Ecology and Management of Cowbirds and Their Hosts*, J. N. M. Smith, T. L. Cook, S. I. Rothstein, S. K. Robinson, S. G. Sealy, Eds. (Univ. of Texas Press, 2000), pp. 69–80.

88. F. Nottebohm, M. E. Nottebohm, Relationship between song repertoire and age in the canary, *Serinus canarius*. *Z. Tierpsychol.* **46**, 298–305 (1978). doi:10.1111/j.1439-0310.1978.tb01451.x

89. H. Williams, Birdsong and singing behavior. *Ann. N. Y. Acad. Sci.* **1016**, 1–30 (2004). doi:10.1196/annals.1298.029 Medline

90. M. Kreutzer, I. Beme, E. Vallet, L. Kiosseva, Social stimulation modulates the use of the "A" phrase in male canary songs. *Behaviour* **136**, 1325–1334 (1999). doi:10.1163/156853999500749

91. E. Vallet, M. Kreutzer, Female canaries are sexually responsive to special song phrases. *Anim. Behav.* **49**, 1603–1610 (1995). doi:10.1016/0003-3472(95)90082-9

92. J. Mulligan, K. Olsen, "Communication in canary courtship calls" in *Bird Vocalisations: Their Relations to Current Problems in Biology and Psychology*, R. A. Hinde, Ed. (Cambridge Univ. Press, 1969).

93. L. S. Putnam, The life history of the cedar waxwing. *Wilson Bull.* **61**, 141–182 (1949).

94. P. Marler, D. Isaac, Physical analysis of a simple bird song as exemplified by the chipping sparrow. *Condor* **62**, 124–135 (1960). doi:10.2307/1365677

95. D. Borror, Songs of the chipping sparrow. *Ohio J. Sci.* **59**, 347 (1959).

96. W. C. Liu, F. Nottebohm, A learning program that ensures prompt and versatile vocal imitation. *Proc. Natl. Acad. Sci. U.S.A.* **104**, 20398–20403 (2007). doi:10.1073/pnas.0710067104 Medline

97. L. H. Walkinshaw, The Eastern chipping sparrow in Michigan. *Wilson Bull.* **56**, 193–205 (1944).

98. D. E. Kroodsma, Development and use of two song forms by the eastern phoebe. *Wilson Bull.* **97**, 21–29 (1985).

99. W. J. Smith, Displays of Sayornis phoebe (Aves, Tyrannidae). *Behaviour* **33**, 283–321 (1969). doi:10.1163/156853969X00125

100. A. C. Bent, *Life histories of North American Flycatchers, Larks, Swallows, and Their Allies. Order Passeriformes (Families Cotingidae, Tyrannidae, Alaudidae, and Hirundinidae)* (United States National Museum Bulletin 179, US Government Printing Office, 1942).

101. M. West, A. Stroud, A. King, Mimicry of the human voice by European starlings: The role of social interaction. *Wilson Bull.* **95**, 635–640 (1983).

102. M. L. Chaiken, J. Böhner, Song learning after isolation in the open-ended learner the European starling: Dissociation of imitation and syntactic development. *Condor* **109**, 968–976 (2007). doi:10.1093/condor/109.4.968

103. M. Goller, thesis, University of Nebraska–Lincoln (2020).

104. E. Hartby, The calls of the starling (*Sturnus vulgaris*). *Dansk Ornithologisk Forenings Tidsskrift* **1969**, 205–230 (1969).

105. A. C. Bent, *Life Histories of North American Nuthatches, Wrens, Thrashers, and Their Allies: Order Passeriformes* (United States National Museum Bulletin 195, US Government Printing Office, 1948).

106. W. L. Thompson, P. L. Jane, An analysis of catbird song. *Jack-Pine Warbler* **47**, 115–125 (1969).

107. D. Rendall, C. D. Kaluthota, Song organization and variability in Northern house wrens (*Troglodytes aedon parkmanii*) in western Canada. *Auk* **130**, 617–628 (2013). doi:10.1525/auk.2013.13069

108. M. E. Platt, M. S. Ficken, Organization of singing in house wrens (Organización del Canto del Reyezuelo (*Troglodytes aedon*)). *J. Field Ornithol.* **58**, 190–197 (1987).

109. M. Fasanella, G. J. Fernández, Alarm calls of the Southern house wren *Troglodytes musculus*: Variation with nesting stage and predator model. *J. Ornithol.* **150**, 853–863 (2009). doi:10.1007/s10336-009-0406-2

110. B. I. Lade, W. H. Thorpe, Dove songs as innately coded patterns of specific behaviour. *Nature* **202**, 366–368 (1964). doi:10.1038/202366a0

111. W. Craig, The expressions of emotion in the pigeons. II. The mourning dove (*Zenaidura macroura* Linn.). *Auk* **28**, 398–407 (1911). doi:10.2307/4071159

112. N. E. Collias, Pigeons and doves of the world. *Auk* **86**, 151–152 (1969). doi:10.2307/4083566

113. L. F. Baptista, W. I. Boarman, P. Kandianidis, Behavior and taxonomic status of Grayson's dove. *Auk* **100**, 907–919 (1983). doi:10.1093/auk/100.4.907

114. R. E. Lemon, Geographic variation in the song of cardinals. *Can. J. Zool.* **44**, 413–428 (1966). doi:10.1139/z66-044

115. R. E. Lemon, The song repertoires of cardinals (*Richmondena cardinalis*) at London, Ontario. *Can. J. Zool.* **43**, 559–569 (1965). doi:10.1139/z65-055

116. R. E. Lemon, C. Chatfield, Organization of song in cardinals. *Anim. Behav.* **19**, 1–17 (1971). doi:10.1016/S0003-3472(71)80129-X

117. R. E. Lemon, The displays and call notes of cardinals. *Can. J. Zool.* **46**, 141–151 (1968). doi:10.1139/z68-023

118. P. Marler, P. Mundinger, M. S. Waser, A. Lutjen, Effects of acoustical stimulation and deprivation on song development in red-winged blackbirds (*Agelaius phoeniceus*). *Anim. Behav.* **20**, 586–606 (1972). doi:10.1016/S0003-3472(72)80024-1 Medline

119. D. E. Kroodsma, F. C. James, Song variation within and among populations of red-winged blackbirds. *Wilson Bull.* **106**, 156–162 (1994).

120. D. G. Smith, F. A. Reid, Roles of the song repertoire in red-winged blackbirds. *Behav. Ecol. Sociobiol.* **5**, 279–290 (1979). doi:10.1007/BF00293676

121. R. W. Nero, Comparative behavior of the yellow-headed blackbird, red-winged blackbird, and other Icterids. *Wilson Bull.* **75**, 376–413 (1963).

122. L. D. Beletsky, B. J. Higgins, G. H. Orians, Communication by changing signals: Call switching in red-winged blackbirds. *Behav. Ecol. Sociobiol.* **18**, 221–229 (1986). doi:10.1007/BF00290826

123. A. A. Ríos-Chelén, G. C. Lee, G. L. Patricelli, A comparison between two ways to measure minimum frequency and an experimental test of vocal plasticity in red-winged blackbirds in response to noise. *Behaviour* **153**, 1445–1472 (2016). doi:10.1163/1568539X-00003390

124. H. G. Kramer, R. E. Lemon, Dynamics of territorial singing between neighboring song sparrows (*Melospiza melodia*). *Behaviour* **85**, 198–223 (1983). doi:10.1163/156853983X00228

125. J. Podos, S. Peters, T. Rudnicky, P. Marler, S. Nowicki, The organization of song repertoires in song sparrows: Themes and variations. *Ethology* **90**, 89–106 (1992). doi:10.1111/j.1439-0310.1992.tb00824.x

126. M. M. Nice, *Studies in the Life History of the Song Sparrow II: The Behavior of the Song Sparrow and Other Passerines*, Transactions of the Linnaean Society of New York Volume VI (The Linnaean Society of New York, ed. 1, 1943).

127. I. Krams, T. Krama, T. M. Freeberg, C. Kullberg, J. R. Lucas, Linking social complexity and vocal complexity: A parid perspective. *Philos. Trans. R. Soc. Lond. B Biol. Sci.* **367**, 1879–1891 (2012). doi:10.1098/rstb.2011.0222 Medline

128. P. K. Gaddis, Differential usage of song types by plain, bridled and tufted titmice. *Ornis Scand.* **14**, 16–23 (1983). doi:10.2307/3676245

129. D. J. Schroeder, R. H. Wiley, Communication with shared song themes in tufted titmice. *Auk* **100**, 414–424 (1983). doi:10.1093/auk/100.2.414

130. P. Gaddis, thesis, University of Florida (1979).

131. J. L. Owens, T. M. Freeberg, Variation in chick-a-dee calls of tufted titmice, *Baeolophus bicolor*: Note type and individual distinctiveness. *J. Acoust. Soc. Am.* **122**, 1216–1226 (2007). doi:10.1121/1.2749459 Medline

132. C. L. Brennan, A. W. Jones, Song structure and cadence of the veery (*Catharus fuscescens*) in the Appalachian Mountains. *Wilson J. Ornithol.* **128**, 75–85 (2016). doi:10.1676/wils-128-01-75-85.1

133. D. M. Weary, R. E. Lemon, E. M. Date, Acoustic features used in song discrimination by the veery. *Ethology* **72**, 199–213 (1986). doi:10.1111/j.1439-0310.1986.tb00621.x

134. C. M. Heckscher, thesis, University of Delaware (2007).

135. G. Ritchison, Vocalizations of the white-breasted nuthatch. *Wilson Bull.* **95**, 440–451 (1983).

136. L. Kilham, Reproductive behavior of white-breasted nuthatches II. Courtship. *Auk* **89**, 115–129 (1972). doi:10.2307/4084063

137. J. B. Falls, "Functions of territorial song in the white-throated sparrow" in *Bird Vocalizations*, R. A. Hinde (Cambridge Univ. Press, 1969), pp. 207–232.

138. D. J. Borror, W. W. H. Gunn, Variation in white-throated sparrow songs. *Auk* **82**, 26–47 (1965). doi:10.2307/4082793

139. J. K. Lowther, J. B. Falls, "White-throated sparrow" in *Life Histories of North American Cardinals, Grosbeaks, Buntings, Towhees, Finches, Sparrows, and Allies, Part Three*, O. L. Austin Jr., Ed. (United States National Museum Bulletin 237, Smithsonian Institution Press, 1968), pp. 1365–1392.

140. D. A. Spector, The singing behaviour of yellow warblers. *Behaviour* **117**, 29–52 (1991). doi:10.1163/156853991X00111

141. K. G. Bankwitz, W. L. Thompson, Song characteristics of the yellow warbler. *Wilson Bull.* **91**, 533–550 (1979).

142. S. A. Gill, thesis, University of Manitoba (1995).

143. J. E. Elie, F. E. Theunissen, The vocal repertoire of the domesticated zebra finch: A data-driven approach to decipher the information-bearing acoustic features of communication signals. *Anim. Cogn.* **19**, 285–315 (2016). doi:10.1007/s10071-015-0933-6 Medline

144. R. Zann, *The Zebra Finch: A Synthesis of Field and Laboratory Studies*, Oxford Ornithology Series (Oxford Univ. Press, 1996).
