# Supplementary material for: Songbird species that display more-complex vocal learning are better problem-solvers and have larger brains
Source: Science. Author manuscript; Available in PMC 2026 Mar 26. (PMC13020620; doi:10.1126/science.adh3428)
Supplement: Audet et al 2023 SOM 2 [file NIHMS1967767-supplement-Audet_et_al_2023_SOM_2.pdf]

Materials Design Analysis Reporting (MDAR) Checklist for Authors

The MDAR framework establishes a minimum set of requirements in transparent reporting applicable to studies in the life sciences (see Statement of Task: doi:10.31222/osf.io/9sm4x.). The MDAR checklist is a tool for authors, editors, and others seeking to adopt the MDAR framework for transparent reporting in manuscripts and other outputs. Please refer to the MDAR Elaboration Document for additional context for the MDAR framework.

For all that apply, please note where in the manuscript the required information is provided.

Materials:

Newly created materials	indicate where provided: page no/section/legend)	n/a
The manuscript includes a dedicated "materials availability statement" providing transparent disclosure about availability of newly created materials including details on how materials can be accessed and describing any restrictions on access.	We created a database of vocal learning features of 23 bird species (table S1), and a behavior dataset based on our observations (doi:10.5061/dryad.tb2rbp06n). The methodology for the creation of this data is provided in details in supplementary materials.	

Antibodies	indicate where provided: page no/section/legend)	n/a
For commercial reagents, provide supplier name, catalogue number and RRID, if available.		x

DNA and RNA sequences	indicate where provided: page no/section/legend)	n/a
**Short novel DNA or RNA including primers, probes:** Sequences should be included or deposited in a public repository.		x

Cell materials	indicate where provided: page no/section/legend	n/a
**Cell lines:** Provide species information, strain. Provide accession number in repository **OR** supplier name, catalog number, clone number, **OR** RRID.		x
**Primary cultures:** Provide species, strain, sex of origin, genetic modification status.		x

Experimental animals	indicate where provided: page no/section/legend)	n/a
**Laboratory animals or Model organisms:** Provide species, strain, sex, age, genetic modification status. Provide accession number in repository **OR** supplier name, catalog number, clone number, **OR** RRID.		x
**Animal observed in or captured from the field:** Provide species, sex, and age where possible.	Individuals of 21 wild bird species were collected in the field: American goldfinch (Spinus tristis), American redstart (Setophaga ruticilla), American robin (Turdus migratorius), Black-capped chickadee (Poecile atricapillus), Blue Jay (Cyanocitta cristata), Brown-headed cowbird (Molothrus ater), Cedar waxwing (Bombycilla cedrorum), Chipping sparrow (Spizella passerina), Eastern phoebe (Sayornis phoebe), European starling (Sturnus vulgaris), Gray catbird (Dumetella carolinensis), House wren (Troglodytes aedon), Mourning dove (Zenaida macroura), Northern cardinal (Cardinalis cardinalis), Red-winged blackbird (Agelaius phoeniceus), Song sparrow (Melospiza melodia), Tufted titmouse (Baeolophus bicolor), Veery (Catharus fuscescens), White-breasted nuthatch (Sitta carolinensis), White-throated sparrow (Zonotrichia albicollis), Yellow warbler (Setophaga petechia). We also tested individuals of two domesticated species: Canary (Serinus canaria) and Zebra finch (Taeniopygia guttata). We tested 207 males and 7 females. All tested wild birds were adults but their age is unknown. Domesticated birds were aged 9-15 months.	

Plants and microbes	indicate where provided: page no/section/legend)	n/a

**Plants:** provide species and strain, ecotype and cultivar where relevant, unique accession number if available, and source (including location for collected wild specimens).		x
**Microbes:** provide species and strain, unique accession number if available, and source.		x

**Human research participants**	**indicate where provided: page no/section/legend) or state if these demographics were not collected**	**n/a**
If collected and within the bounds of privacy constraints report on age, sex and gender or ethnicity for all study participants.		x

Design:

Study protocol	indicate where provided: page no/section/legend)	n/a
If study protocol has been pre-registered, provide DOI. For clinical trials, provide the trial registration number **OR** cite DOI.		x

Laboratory protocol	indicate where provided: page no/section/legend)	n/a
Provide DOI **OR** other citation details if detailed step-by-step protocols are available.		x

Experimental study design (statistics details)		
**For in vivo studies:** State whether and how the following have been done	**indicate where provided: page no/section/legend. If it could have been done, but was not, write not done**	**n/a**
Sample size determination	We tested a sufficient number of individuals per species to allow for statistical analyses (mean n per species = 9.3). The choice of species is explained in detail in supplementary materials pages 2 and 10.	
Randomisation	The birds were tested in order of their capture. The behavioral tasks were not randomized since the test order is expected to strongly influence the performance; therefore, it was kept identical for all tested birds.	
Blinding	During the behavioral tests, the experimenter did not have prior knowledge on the cognitive capacities and brain sizes of the tested species. The behavioral data were only analyzed at the end of the field seasons; therefore, the results were unknown throughout the testing period.	
Inclusion/exclusion criteria	We included all available data on the 23 species.	

Sample definition and in-laboratory replication	indicate where provided: page no/section/legend	n/a
State number of times the experiment was replicated in laboratory.	The whole behavioral protocol was given to each 214 individuals (replication: on average 9.3 times per species).	
Define whether data describe technical or biological replicates.	Biological.	

Ethics	indicate where provided: page no/section/legend	n/a
**Studies involving human participants:** State details of authority granting ethics approval (IRB or equivalent committee(s), provide reference number for approval.		x
**Studies involving experimental animals:** State details of authority granting ethics approval (IRB or equivalent committee(s), provide reference number for approval.	The study was conducted in compliance with all local and national regulations. Permits were issued by Rockefeller University (IACUC permit # 17084), New York State Department of Environmental Conservation (Banding permit # 198, Scientific collection permit # 2284), United States Fish and Wildlife Service (Permit # MB-45822C) and United States Geological Survey (Permit # 24130).	
**Studies involving specimen and field samples:** State if relevant permits obtained, provide details of authority approving study; if none were required, explain why.		x

Dual Use Research of Concern (DURC)	indicate where provided: page no/section/legend	n/a

If study is subject to dual use research of concern regulations, state the authority granting approval and reference number for the regulatory approval.		x

Analysis:

Attrition	indicate where provided: page no/section/legend	n/a
Describe whether exclusion criteria were preestablished. Report if sample or data points were omitted from analysis. If yes report if this was due to attrition or intentional exclusion and provide justification.		x

Statistics	indicate where provided: page no/section/legend	n/a
Describe statistical tests used and justify choice of tests.	Statistical analyses are described in supplementary materials, pages 8-9.	

Data availability	indicate where provided: page no/section/legend	n/a
For newly created and reused datasets, the manuscript includes a data availability statement that provides details for access or notes restrictions on access.	The vocal learning features dataset is provided in table S1. The behavioral data is available at doi:10.5061/dryad.tb2rbp06n	
If newly created datasets are publicly available, provide accession number in repository **OR** DOI **OR** URL and licensing details where available.		x
If reused data is publicly available provide accession number in repository **OR** DOI **OR** URL, **OR** citation.	We used a published brain size database (Cited in-text: Sayol et al., 2018).	

Code availability	indicate where provided: page no/section/legend	n/a
For all newly generated custom computer code/software/mathematical algorithm or re-used code essential for replicating the main findings of the study, the manuscript includes a data availability statement that provides details for access or notes restrictions.	The R code is available at doi:10.5061/dryad.tb2rbp06n	
If newly generated code is publicly available, provide accession number in repository, **OR** DOI **OR** URL and licensing details where available. State any restrictions on code availability or accessibility.		x
If reused code is publicly available provide accession number in repository **OR** DOI **OR** URL, **OR** citation.		x

Reporting

MDAR framework recommends adoption of discipline-specific guidelines, established and endorsed through community initiatives. Journals have their own policy about requiring specific guidelines and recommendations to complement MDAR.

Adherence to community standards	indicate where provided: page no/section/legend	n/a
State if relevant guidelines (e.g., ICMJE, MIBBI, ARRIVE) have been followed, and whether a checklist (e.g., CONSORT, PRISMA, ARRIVE) is provided with the manuscript.		x
